# A Novel System of Polymorphic and Diverse NK Cell Receptors in Primates

**DOI:** 10.1371/journal.pgen.1000688

**Published:** 2009-10-16

**Authors:** Anne Averdam, Beatrix Petersen, Cornelia Rosner, Jennifer Neff, Christian Roos, Manfred Eberle, Fabienne Aujard, Claudia Münch, Werner Schempp, Mary Carrington, Takashi Shiina, Hidetoshi Inoko, Florian Knaust, Penny Coggill, Harminder Sehra, Stephan Beck, Laurent Abi-Rached, Richard Reinhardt, Lutz Walter

**Affiliations:** 1 Department of Primate Genetics, German Primate Centre, Göttingen, Germany; 2 Gene Bank of Primates, German Primate Centre, Göttingen, Germany; 3 Department of Behavioural Ecology and Sociobiology, German Primate Centre, Göttingen, Germany; 4 Adaptive Mechanisms and Evolution, UMR CNRS/MNHN 7179, Brunoy, France; 5 Institute for Human Genetics, University of Freiburg, Freiburg, Germany; 6 Cancer and Inflammation Program, Laboratory of Experimental Immunology, SAIC-Frederick, Inc., National Cancer Institute-Frederick, Frederick, Maryland, United States of America; 7 Tokai University School of Medicine, Isehara, Japan; 8 Max-Planck-Institute for Molecular Genetics, Berlin, Germany; 9 Wellcome Trust Sanger Institute, Hinxton, United Kingdom; 10 University College London Cancer Institute, University College London, London, United Kingdom; 11 Department of Structural Biology, Stanford University School of Medicine, Stanford, California, United States of America; Fred Hutchinson Cancer Research Center, United States of America

## Abstract

There are two main classes of natural killer (NK) cell receptors in mammals, the killer cell immunoglobulin-like receptors (KIR) and the structurally unrelated killer cell lectin-like receptors (KLR). While KIR represent the most diverse group of NK receptors in all primates studied to date, including humans, apes, and Old and New World monkeys, KLR represent the functional equivalent in rodents. Here, we report a first digression from this rule in lemurs, where the KLR (CD94/NKG2) rather than KIR constitute the most diverse group of NK cell receptors. We demonstrate that natural selection contributed to such diversification in lemurs and particularly targeted KLR residues interacting with the peptide presented by MHC class I ligands. We further show that lemurs lack a strict ortholog or functional equivalent of MHC-E, the ligands of non-polymorphic KLR in “higher” primates. Our data support the existence of a hitherto unknown system of polymorphic and diverse NK cell receptors in primates and of combinatorial diversity as a novel mechanism to increase NK cell receptor repertoire.

## Author Summary

Most receptors of natural killer (NK) cells interact with highly polymorphic major histocompatibility complex (MHC) class I molecules and thereby regulate the activity of NK cells against infected or malignant target cells. Whereas humans, apes, and Old and New World monkeys use the family of killer cell immunoglobulin-like receptors (KIR) as highly diverse NK cell receptors, this function is performed in rodents by the diverse family of lectin-like receptors Ly49. When did this functional separation occur in evolution? We followed this by investigating lemurs, primates that are distantly related to humans. We show here that lemurs employ the CD94/NKG2 family as their highly diversified NK cell receptors. The CD94/NKG2 receptors also belong to the lectin-like receptor family, but are rather conserved in “higher” primates and rodents. We could further demonstrate that lemurs have a single *Ly49* gene like other primates but lack functional *KIR* genes of the KIR3DL lineage and show major deviations in their MHC class I genomic organisation. Thus, lemurs have evolved a “third way” of polymorphic and diverse NK cell receptors. In addition, the multiplied lemur CD94/NKG2 receptors can be freely combined, thereby forming diverse receptors. This is, therefore, the first description of some combinatorial diversity of NK cell receptors.

## Introduction

Natural killer (NK) cells are bone marrow-derived lymphocytes that form an essential part of the immune response against pathogens and are involved in the elimination of tumour cells. Equipped with a diverse array of germline-encoded receptors that are able to mediate inhibitory or activating signals [1], NK cells scan other cells for the presence of ligands of these receptors [2]. Activation of NK cells is typically achieved by discontinuation of inhibitory signalling and involvement of activating receptors, resulting in cytokine release or killing of target cells [2]–[4]. Most NK cell receptors interact with members of the MHC class I protein family and either belong to the killer cell lectin-like receptors (KLR) of the C-type lectin-like family such as CD94, NKG2, or Ly49, or the killer cell immunoglobulin-like (KIR) receptors, which are encoded in the natural killer complex (*NKC*) and leukocyte receptor complex (*LRC*), respectively [1]. Both *NKC* and *LRC* contain inhibitory and activating NK cell receptors. Inhibitory receptors are characterised by the presence of immunoreceptor tyrosine-based inhibitory motifs (ITIM) in the cytoplasmic tail, whereas activating receptors lack ITIMs and instead contain a positively charged amino acid (arginine or lysine) in their transmembrane region, thereby associating with signalling adaptor molecules DAP10, DAP12 or FcγR [2].

The polymorphic NK cell receptors are represented by KIR in humans, apes, Old World and New World monkeys [5]–[7] and KLR (Ly49 molecules) in rodents [8]. These two receptor systems are not structurally related, but have similar functions: interaction with MHC class I molecules and regulation of NK cell activity [2]. Due to these functional constraints, *KIR* and *Ly49* genes have to keep pace with the rapidly evolving and polymorphic class I genes [9] and it has been shown that combinations of this highly complex and polymorphic genetic system of NK cell receptors and their MHC class I ligands can significantly influence susceptibility and resistance to infectious and malignant diseases, autoimmune disorders, and reproduction [10]. In contrast to *KIR* and *Ly49*, another genetic system of MHC class I-binding NK cell receptors has been kept conserved in primates (hominoids, Old World and New World monkeys) [11] and rodents [12], the CD94 and NKG2 molecules, which also belong to the KLR family. The CD94 molecule can either pair with the inhibitory NKG2A or the activating NKG2C and NKG2E molecules and these heterodimeric NK cell receptors specifically recognise conserved nonclassical class I molecules, HLA-E in humans [13] and H2-Qa1 in mice [14]. These nonclassical class I molecules bind peptides derived from signal sequences of certain class I molecules [15], thereby monitoring putative downregulation of the corresponding class I molecules mediated by pathogens as part of their immune evasion strategy.

Various NK cell receptors (CD94/NKG2, KIR, Ly49L) have been identified in different nonhuman primates, and rapid evolution in particular of the *KIR* genes was reported [16]. Yet, two questions remain open: how far can we trace back *KIR3DL* diversification and what was the NK cell receptor content in the ancestor of all primates? We report here that strepsirrhine primates such as lemurs have evolved a ‘third way’ of a diverse NK cell receptor system and exhibit highly diversified, positively selected CD94/NKG2 receptors. This NK cell receptor system is further characterised by combinatorial diversity.

## Results

In the absence of a formal taxonomic designation, we refer to strepsirrhines (lemurs, galagos, lorises) and tarsier as ‘lower’ primates and to humans, apes, Old and New World monkeys as ‘higher’ primates throughout the manuscript.

### Unusual organisation of the *LRC*, *NKC*, and *MHC* in “lower” primates

**Figure 1:**
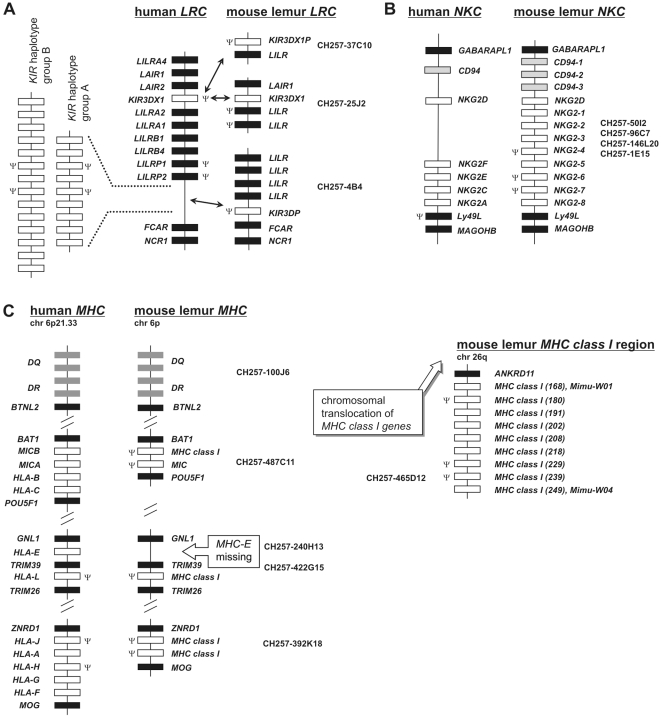
Comparison of human and grey mouse lemur LRC, NKC, and MHC genomic regions. Ψ denotes pseudogene (criteria based on: premature stop codons, frameshift deletions/insertions, absence of essential coding/non-coding parts). (A) Sequenced BAC clones (GenBank accession no. CR974412, CR974436, CR974413) of the LRC. Open rectangles denote KIR genes, filled rectangles all other genes. Arrows point to corresponding KIR genes. No gene designations are shown for human KIR haplotypes. (B) Sequenced BAC clone contig of the NKC that constitute a complete haplotype. Open rectangles denote NKG2 genes, grey rectangles CD94 genes, and filled rectangles all other genes. The order of BAC clones in this CD94 to Ly49L genomic interval is: 50I2, 96C7, 146L20, and 1E15 (haplotype 1: GenBank accession no. FP236838). A second contig of BAC clones includes the CD94-1-NKG2-8 interval represented by clones 481C2, 492D19, 489H5, and 222F7 (not shown) and constitutes a second haplotype (GenBank accession no. FP236834). (C) Sequenced BAC clones (GenBank accession no. AB480748, FP236831, FP236832, FP236833, FP236839) of the MHC class I gene regions. Class I genes of BAC clone CH257-465D12 that could not be assigned to an already known gene or allele, are named according to their position on the BAC clone (numbers in parentheses). Putative alleles Mimu-W01 and Mimu-W04 are indicated. Open rectangles denote MHC class I genes, grey rectangles MHC class II genes, and filled rectangles all other genes.

Clones containing the *LRC*, *NKC*, and *MHC* genomic regions were isolated from a BAC library of the grey mouse lemur (*Microcebus murinus*). Nineteen BAC clones from the *LRC* region were identified and three clones covering the *KIR-LILR* subregion were sequenced: one clone contains the *NCR1*, *FCAR*, and *LILR* genes in addition to a *KIR3DP* pseudogene mapping between *FCAR* and the *LILR* gene cluster, and the other two clones both include *LILR* genes and a single copy of the *KIR3DX1* [17] gene (Figure 1A). Similar to all other primate *KIR3DX1* genes, the two lemur copies, of which only one is intact, also lack the *MLTD1* repetitive elements in intron 3. The intact copy is transcribed in lemur PBMC and alternatively spliced (Figure S1); the other locus (*KIR3DX1P*) is a fragmented pseudogene. In contrast, *KIR3DP* is characterised by the presence of *KIR3DL*-typical repetitive elements such as *MLT1D/LTR33A*, *MSTB1*, *MER70B* [7] and is thus the only representative of the *KIR3DL* lineage in the grey mouse lemur. The repetitive elements flanking *KIR3DP* are found in the ‘outer’ human *KIR* genes *KIR3DL3* and *KIR3DL2* (data not shown), suggesting that the lemur *KIR3DP* is equivalent to all ‘higher’ primate *KIR* genes. Thus, the only functional *KIR* in the mouse lemur genome is represented by *KIR3DX1*, a *KIR* gene of unknown function [17].

Unlike the *KIR* region, the *CD94*-*Ly49L* genomic interval of the grey mouse lemur *NKC* showed amplifications of the *CD94* and *NKG2* genes (Figure 1B), which accounted for its 1.5 times increased size compared to humans. Only two genes in this region represent one-to-one orthologs of ‘higher’ primate genes: *NKG2D* and *Ly49L*. Further investigations of the three mouse lemur *CD94* genes indicate they encode typical CD94 receptor structures but their amino acid sequences show considerable diversity, differing by 23–24% among each other (Figure S2). Such level of divergence is significantly higher than in ‘higher’ primates, as human and common marmoset monkey CD94 sequences differ only by 13% for example [11]. Furthermore, eight *NKG2*-related genes were identified (Figure 1B), of which five are functional genes (Figure S3) and three are pseudogenes. The NKG2 molecules show different combinations of functionally relevant motifs: ITIM, positively charged residue in the transmembrane region, and the YxxM motif, which is a recognition site for the p85 subunit of phosphatidylinositol 3-kinase (PI3K). Allelic substitutions affect the presence of ITIM and YxxM motifs in *NKG2-2* and *NKG2-3* (Figure S3). These findings suggest that the mouse lemur NKG2 receptors are functionally complex, with inhibitory and activating properties.

**Figure 2:**
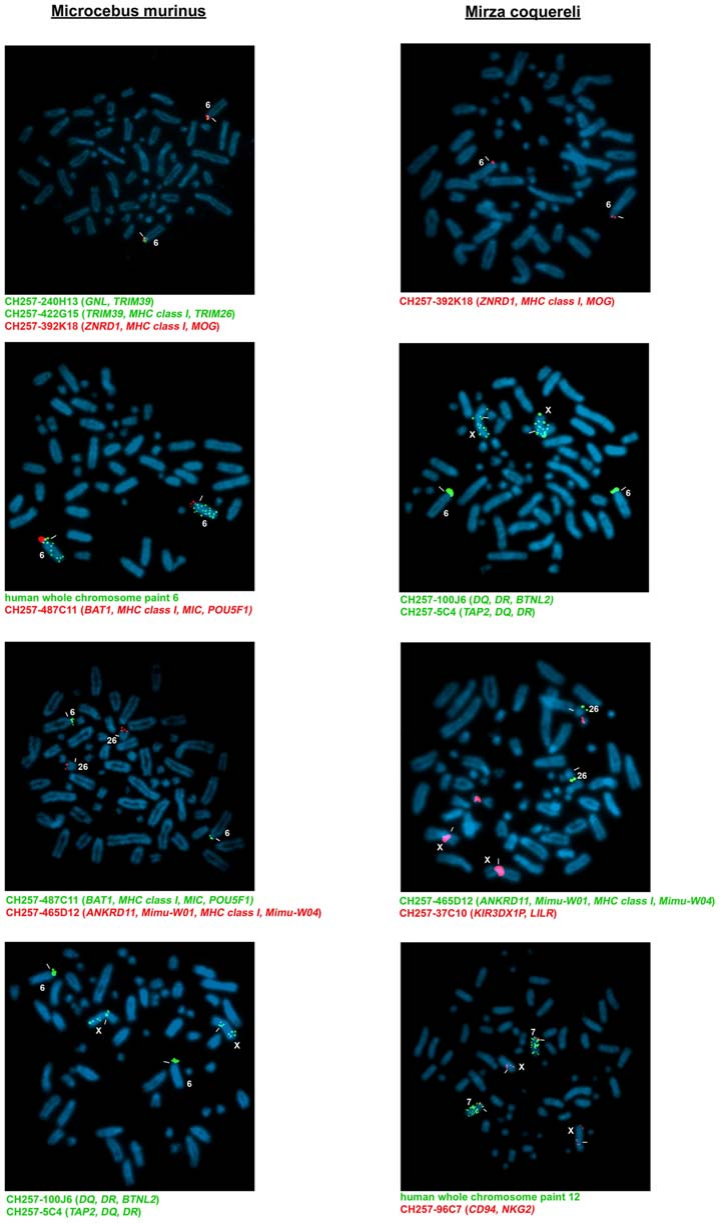
Chromosomal localisation of lemur MHC and NK cell receptor genes. FISH using mouse lemur BACs containing MHC class I and class II genes reveals a split of class I genes to chromosome 6 (GNL, TRIM39, TRIM26, ZNRD1, MOG, BAT1, MIC, POU5F1) and to chromosome 26 (ANKRD11, Mimu-W01, Mimu-W04) of Microcebus murinus. Nomenclature of M.murinus and M.coquereli chromosomes are according to Ref. [43] The same split is shown for Mirza coquereli: ZNRD1 and MOG hybridise to chromosome 6 and ANKRD11, Mimu-W01, and Mimu-W04 to chromosome 26. All tested MHC class II region genes (TAP2, DQ, DR, BTNL2) map to chromosome 6 in Microcebus murinus and Mirza coquereli, respectively. Note that in both species secondary signals appear scattered along the X chromosome. In Mirza coquereli, KIR-including BAC CD257-37C10 hybridises to a small acrocentric chromosome not identical with chromosome 26 and to the X chromosome, and NKG2-including BAC CH257-96C7 maps to chromosome 7, which is orthologous to human chromosome 12. Note that further signals appear on the chromosome X.

BAC clones from the *MHC class I* and *class II* regions could be mapped to the short arm of mouse lemur chromosome 6 by fluorescence in-situ hybridisation (FISH) (Figure 2). Whereas the *class II* region is conserved (Averdam et al., manuscript in preparation), the linked *class I* gene-containing regions of the mouse lemur *MHC* lack any functional *class I* genes as the four identified *class I* genes and the single *MIC* gene all represent pseudogenes (Figure 1C). Remarkably, even *MHC-E and MHC-F*, the only two conserved *MHC class I* genes of ‘higher’ primates, are missing in the mouse lemur *MHC*. Interestingly, our screening of the mouse lemur BAC library for *MHC class I* genes also identified an unlinked genomic region that includes nine *MHC class I* genes. Complete sequencing of this region revealed six genes encoding functional MHC class I proteins, including putative alleles of the previously described classical *class I* genes of the mouse lemur, *Mimu-W01* and *Mimu-W04* [18] (Figure 1C). This *class I* gene cluster maps to another chromosome, the long arm of mouse lemur chromosome 26 (Figure 2), thus providing an additional example of a mammalian species where *MHC class I* and *class II* genes are not linked. A similar chromosomal splitting of *class I* genes was found for a further lemur species, Coquerel's giant mouse lemur (*Mirza coquereli*) (Figure 2). The *CD94* and *NKG2* genes could be mapped to chromosome 7 in the giant mouse lemur, which corresponds to human chromosome 12, indicating conserved synteny of the *NKC* region. The *KIR3DX1P* gene-containing BAC was mapped to a small acrocentric chromosome not identical with chromosome 26 (Figure 2).

### Evolution of *CD94*/*NKG2* and *MHC class I* genes in “lower” and “higher” primates

Using the mouse lemur *NKC* sequences as reference we investigated corresponding cDNA sequences from the black-and-white ruffed lemur (*Varecia variegata*), a species that diverged from the grey mouse lemur about 43 million years ago (mya) [19]. A single *NKG2D*, five *CD94*, nine *NKG2* and two *Ly49L* cDNA sequences were isolated from ruffed lemur PBMC by RT-PCR cloning. The deduced amino acid sequences exhibit similar functional features as described above for mouse lemur CD94 and NKG2 molecules (Figure S2, Figure S3). Two pairs of *CD94* sequences (CD94-2*01, CD94-2*02, and CD94-3*01, CD94-3*02) only differ by a few nucleotide substitutions and are, therefore, regarded as alleles. Similarly, two allelic *NKG2* (NKG2-6*01, NKG2-6*02) and two allelic *Ly49L* (Ly49L*01, Ly49L*02) sequences were isolated, so that the ruffed lemur *NKC* is estimated to possess three *CD94*, eight *NKG2* genes and single *NKG2D* and *Ly49L* genes.

**Figure 3:**
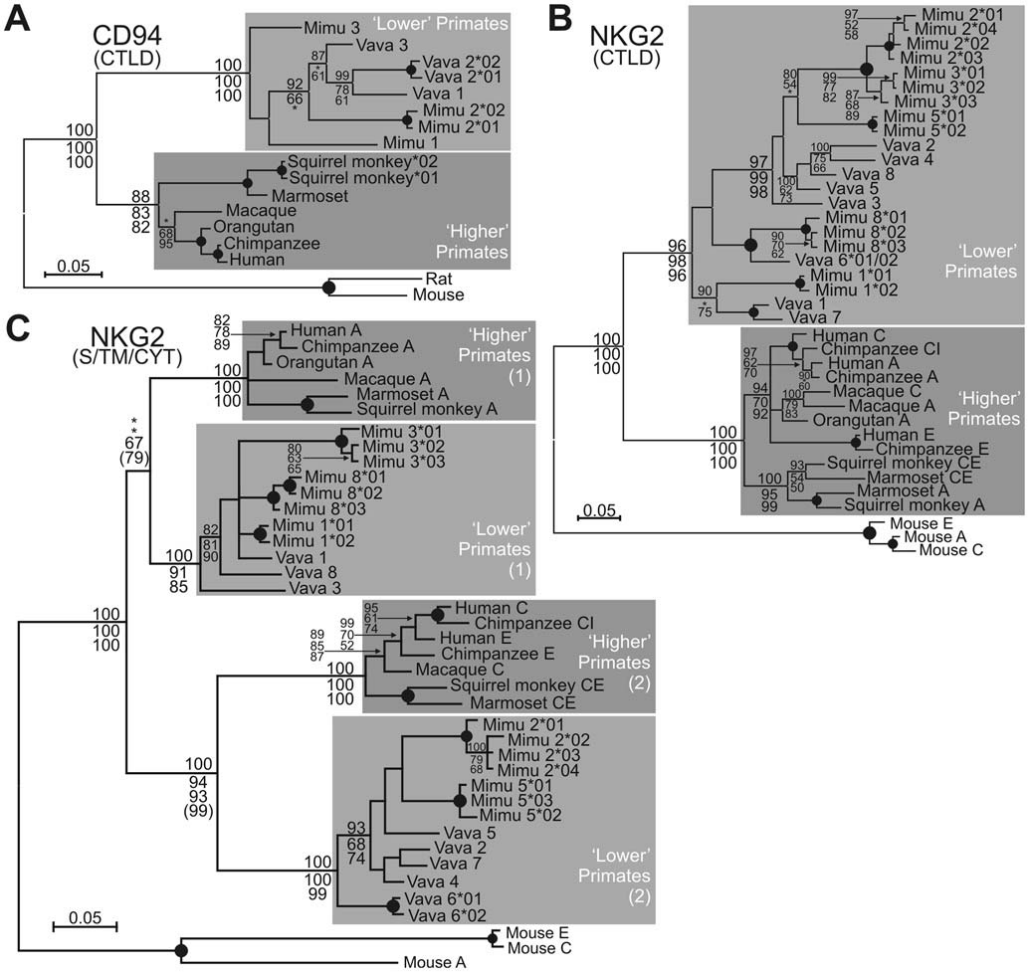
Major diversification of CD94 and NKG2 occurred in “lower” primates after their separation with “higher” primates. Phylogenetic analyses of primate CD94 (A) and NKG2 (B) C-type lectin-like domain sequences and NKG2 stalk, transmembrane, cytoplasmic regions (C). The tree topology obtained with the Neighbor-Joining (NJ) analysis was used for the display (with a midpoint rooting) and numbers at nodes indicate support obtained for Bayesian, parsimony, and NJ methods (from top to bottom). Support is shown if Bayesian posterior probability (PP) ≥ 88% and other methods bootstrap proportion (BP) ≥ 50% (at least 2 methods). Filled circles at nodes indicate PP > 95% and BP ≥ 80%. *: BP < 50 or PP < 80. Mimu, Microcebus murinus; Vava, Varecia variegata. A maximum-likelihood analysis was also performed, and the maximum-likelihood bootstrap support is indicated in parenthesis for two nodes.

Phylogenetic analyses of primate *CD94* and *NKG2* sequences encoding the C-type lectin-like domain indicate that for both gene families ‘lower’ and ‘higher’ primate sequences form their own groups, indicating that diversification of *CD94* and *NKG2* occurred in ‘lower’ primates after their separation with ‘higher’ primates (Figures 3A and 3B). ‘Lower’ primate *NKG2* sequences form three groups, each with sequences from both lemurs, pointing to duplications that preceded speciation of both lemurs (Figure 3B). Similarly, in the largest of the three ‘lower’ primate NKG2 groups, some gene sequences show species-specific clustering indicating gene duplications following speciation of both lemurs. Such patterns demonstrate that *NKG2* gene duplications in ‘lower’ primates occurred both before and after the separation of the two lemur species, a process similar to that seen for *KIR* in ‘higher’ primates and *Ly49* in rodents [20]. In contrast to the C-type lectin-like domain, *NKG2* sequences encoding the stem, cytoplasmic, and transmembrane part fall into two branches: one contains ‘lower’ and ‘higher’ primate inhibitory *NKG2*, the other one containing ‘lower’ and ‘higher’ primate activating *NKG2*, indicating that these two types of sequences separated before the speciation between ‘higher’ and ‘lower’ primates (Figure 3C).

To determine whether amplification of *CD94* genes is restricted to strepsirrhine primates living in Madagascar (lemurs) or is common to other ‘lower’ primates, we investigated the *CD94* sequences of an African and an Asian primate, the potto (*Perodicticus potto*) and the tarsier (*Tarsius syrichta*). Whereas the potto belongs to the primate suborder Strepsirrhini, the tarsier is a member of the other primate suborder, the Haplorrhini (tarsiers, New and Old World monkeys, apes and human). Using all available primate sequences as reference, generic primers were constructed to amplify the region including exon 4, intron 4 and exon 5 of *CD94* and we characterised seven and nine different *CD94* sequences in potto and tarsier, respectively (Figure S4). These findings thus indicate that *CD94* gene amplification is not restricted to lemurs, but can be found in other strepsirrhine primates and even in a primate more closely related to humans than to lemurs [21]. Insertions of repetitive elements of the *Alu* family in intron 4 revealed additional information: whereas none of the three mouse lemur *CD94* genes contains any *Alu* element, four of the seven potto sequences include an *AluJo* and all nine tarsier sequences have an *AluJb* element (Figure S4). However, the integration sites of these *Alu* elements differ between potto and tarsier, indicating that integration events occurred after these lineages diverged from each other. Hence, the amplification of *CD94* sequences occurred repeatedly and independently in ‘lower’ primates. The intron 4 sequences of human, rhesus macaque and common marmoset *CD94* share an *AluSx* integration (data not shown), which is not present in ‘lower’ primates, further supporting the monophyletic origin of ‘higher’ primate *CD94*.

**Figure 4:**
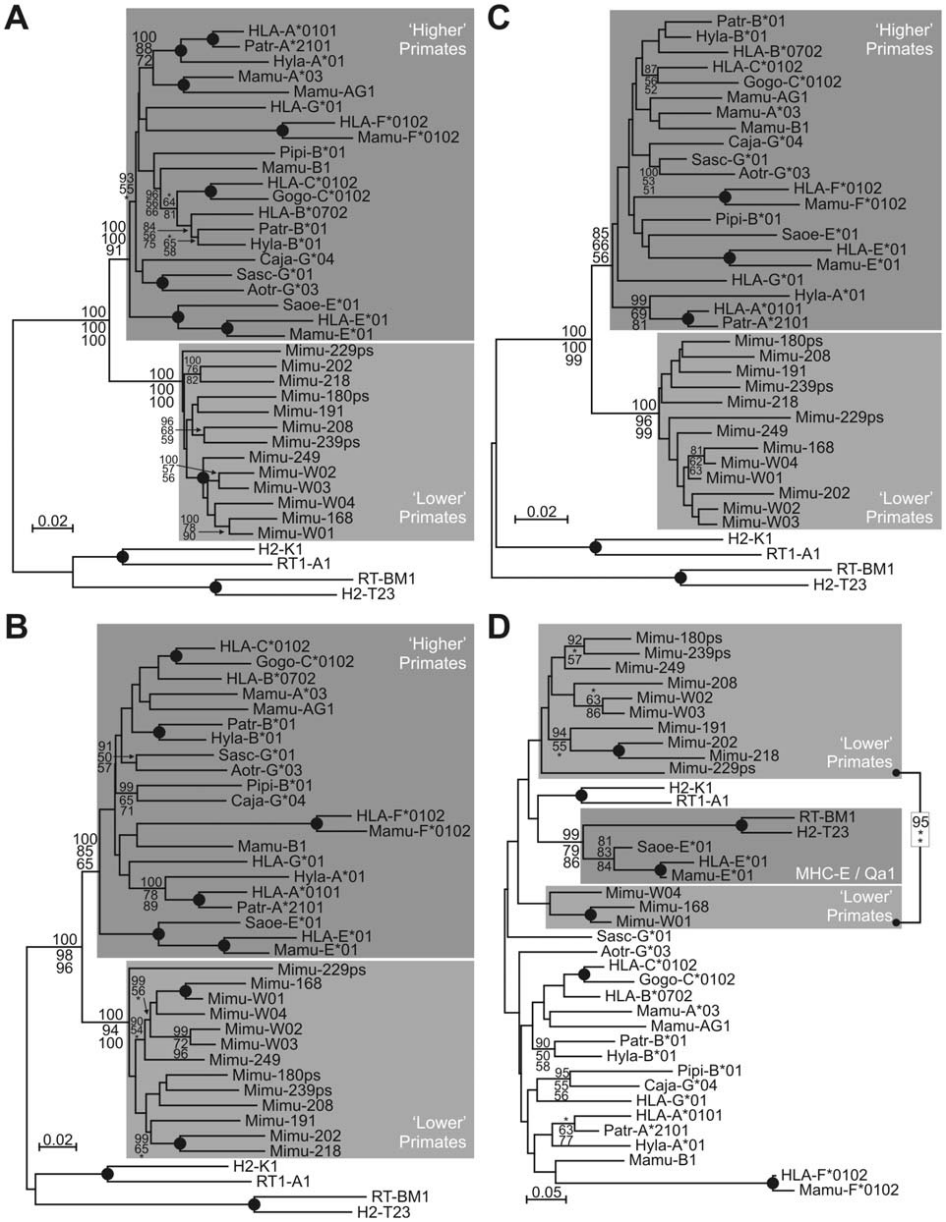
“Lower” primates lack a strict ortholog or functional equivalent of “higher” primate MHC-E. Phylogenetic analyses of MHC class I genes with complete coding sequences (A), peptide binding domain (B), peptide binding domain excluding peptide binding residues (C) and peptide binding residues only (D). A phylogenetic analysis of MHC class I genes with complete coding sequences excluding the peptide binding residues was also conducted and the results are presented in Figure S5. Analysis and display are as described for Figure 3. Numbers as gene names for MHC class I are the same as in Figure 1C. Non-primate sequences included are mouse (H2) and rat (RT1). Peptide binding residues were defined according to Bjorkman et al. [44]. Patr, Pan troglodytes; Gogo, Gorilla gorilla; Hyla, Hylobates lar; Saoe, Saguinus oedipus; Sasc, Saimiri sciureus; Mamu, Macaca mulatta; Mimu, Microcebus murinus; Aotr, Aotus trivirgatus; Caja, Callithrix jacchus; Pipi, Pithecia pithecia.

While lemurs (and possibly other ‘lower’ primates) experienced a diversification of the CD94/NKG2 receptor system, they appear to lack MHC-E, the ligand of CD94/NKG2 in ‘higher’ primates, as their *MHC* region lacks a *class I* gene where the *MHC-E* gene of ‘higher’ primates is located (Figure 1C). To investigate if any of the grey mouse lemur *MHC class I* genes represents an ortholog or functional equivalent of ‘higher’ primate *MHC-E*, we performed phylogenetic analyses of primate *MHC class I* sequences. Analyses of the full-length sequences and of the peptide-binding-domain (PBD) alone, with or without the peptide binding residues (PBR) indicate that ‘lower’ and ‘higher’ primate sequences form their own groups (Figure 4A–4C, Figure S5). This demonstrates that *MHC class I* diversification in ‘lower’ and ‘higher’ primates took place in each taxonomic lineage after their separation and further indicates that the duplication that gave rise to the *MHC class I* genes on chromosome 26q occurred in the ‘lower’ primate lineage. Similarly, analysis of the PBR revealed that the ‘higher’ primate MHC-E sequences are more closely related to the rodent Qa1 group than to any of the ‘lower’ primate sequences (Figure 4D). Thus, these findings indicate that the grey mouse lemur genome neither encodes a strict orthologue (with a one-to-one relationship) nor a functional homologue of *MHC-E* and confirm earlier data of our group that ‘higher’ and ‘lower’ primate *MHC class I* genes lack strict orthology [18].

### *NKC*-encoded genes of “lower” primates are genetically and functionally diverse

**Figure 5:**
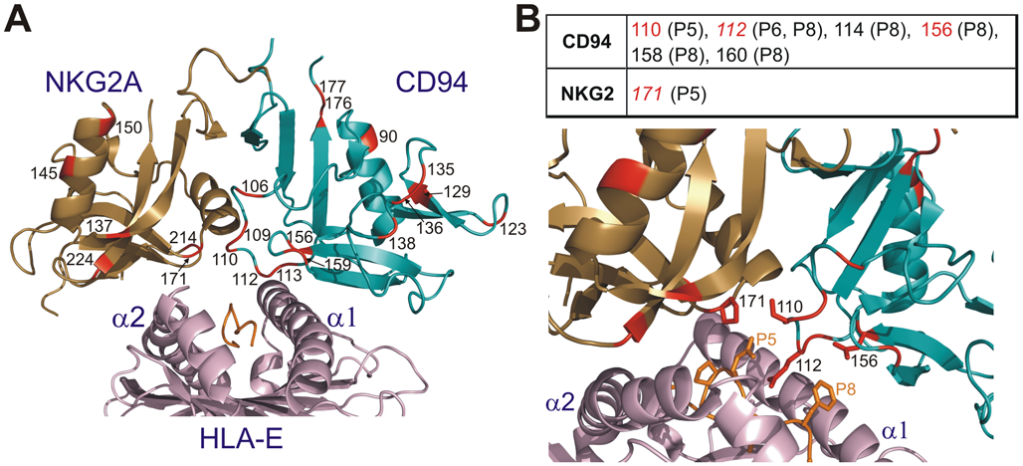
Natural selection diversified “lower” primate CD94 and NKG2 sequences and particularly targeted the residues interacting with the peptide presented by MHC class I ligands. (A) Positively selected sites of ‘lower’ primates CD94/NKG2 superimposed on the three-dimensional structure of the human CD94/NKG2A/HLA-E complex [22],[23]. The PDB file 3cdg was used and represented with PyMOL [45]. (B) Top panel: CD94/NKG2 residues known to interact with MHC class I-bound peptide (based on human CD94/NKG2A/HLA-E). Positively selected residues in ‘lower’ primates are shown in red. Peptide positions are indicated. Residue in italics indicates allelic substitutions were found at this site. Bottom panel: ribbon diagram focused on the region where the interaction between CD94-NKG2A and HLA-E occurs; side chains are displayed.

Our genomic analysis in one mouse lemur individual and cDNA sequencing in three individuals (two mouse lemurs and one black-and-white ruffed lemur) revealed that ‘lower’ primates experienced a diversification of both *CD94* and *NKG2*. Such diversification is in contrast with the situation in ‘higher’ primates where *CD94* is a non-polymorphic, single-copy gene and where the *NKG2* gene family experienced limited diversification with moderate polymorphism. To investigate if such difference is the result of natural selection favouring functional diversity in ‘lower’ but not in ‘higher’ primates, we compared the non-synonymous to synonymous substitution ratio rate (dN/dS) of *CD94* and *NKG2* sequences of ‘lower’ and ‘higher’ primates (Table 1). This analysis shows that ‘lower’ primate *CD94* sequences display highly significant evidence of positive diversifying selection (α = 0.001) with 15 positions positively selected (posterior probability (PP)>0.95). In contrast, their ‘higher’ primate counterparts do not show any evidence of positive selection (Table 1). A similar, yet not as marked, trend is seen for *NKG2* as ‘lower’ primate sequences show highly significant evidence of positive diversifying selection (α = 0.001), with six positions being positively selected (PP>0.95) while in ‘higher’ primates the evidence was not as strong (α = 0.01–0.05) and limited to only one site (Table 1). Mapping of the 21 positively selected sites in ‘lower’ primates on the three-dimensional model of the human CD94/NKG2A heterodimer in contact with HLA-E [22],[23] suggests that many of these positions represent functionally relevant sites (Figure 5A). Indeed, closer inspection revealed that the distribution of these 21 positively selected sites in the *CD94*/*NKG2* lectin-like domains shows a significant bias (α = 0.05) toward the 54 sites involved in ligand-binding and/or dimer formation (Table 2, Figure S6). Further dissection of this bias shows it is particularly marked for the 28 ligand-binding sites (α = 0.01), but not for the 30 sites involved in dimer formation. Within the ligand-binding sites a significant bias toward the 7 sites contacting the MHC class I peptide (α = 0.003) was observed, but for the sites contacting the MHC-class I heavy chain the bias was marginal (α∼0.06) (Table 2, Figure S6). Consistent with this, the four CD94/NKG2 positively selected positions that contact the MHC class I peptide account for 75% (9 out of 12) of all the contacts with the peptide (Figure 5B). This analysis thus shows that the genomic diversification of *CD94*/*NKG2* in ‘lower’ primates was accompanied by positive diversifying selection that particularly targeted the sites contacting the MHC class I peptide. In ‘higher’ primates the limited (NKG2) or lack of (CD94) genomic diversification was accompanied by limited (NKG2) or lack of (CD94) sequence diversification through positive diversifying selection.

**Table 1:** Natural selection diversified CD94 and NKG2 residues in “lower” primates, but not in “higher” primates.

		Comparison	2Δl	α	Proportion of selected sites	dN/dS for selected sites	Positively selected sites (M8 p>0.95)
CD94	‘Lower’ primates	M1a vs. M2a	37.66	0.001	0.26	5.51	
		M8a vs. M8	37.66	0.001	0.26	5.46	***90***, ***106***, 109, 110, ***112***, ***113***, ***123***, 129, *135*, *136*, **138**, **156**, 159, *176*, 177
		M7 vs. M8	37.74	0.001	0.26	5.46	(same as M8a vs. M8)
	‘Higher’ primates	M1a vs. M2a	2.29	NS	**	**	**
		M8a vs. M8	2.29	NS	**	**	**
		M7 vs. M8	3.05	NS	**	**	**
NKG2	‘Lower’ primates	M1a vs. M2a	16.98	0.001	0.15	2.72	
		M8a vs. M8	17.45	0.001	0.19	2.50	***137***, *145*, ***150***, *171*, 214, *224*
		M7 vs. M8	24.73	0.001	0.19	2.50	(same as M8a vs. M8)
	‘Higher’ primates	M1a vs. M2a	7.81	0.05	0.07	3.18	
		M8a vs. M8	7.43	0.01	0.14	2.52	**168**
		M7 vs. M8	10.02	0.01	0.14	2.52	(same as M8a vs. M8)

Maximum likelihood estimation of dN/dS ratios and detection of positively selected positions for the NKG2 and CD94 lectin-like domain sequences of ‘higher’ and ‘lower’ primates. The results of the likelihood ratio tests (see methods) are indicated on the left side of the panel and the positions detected as positively selected (with model M8) are listed on the right side of the panel (p>0.95; p>0.99 shown in bold). α, error level to reject the null model that does not allow for positive selection. NS, not significant. **, Bayesian analysis was not performed when the likelihood ratio tests were not significant. Residues in italics denote allelic substitutions were found at this site. Amino acid positions and numbering refer to human CD94 and NKG2A sequences and equivalent positions in aligned non-human primate sequences.

**Table 2:** Natural selection targeted the CD94/NKG2 residues interacting with the peptide presented by MHC class I ligands in “lower” primates.

	All	Ligand binding	Dimer	Peptide	MHC
Total Selected Sites	21	21	21	21	21
Selected Sites in Category	9	7	4	4	5
Total Residues (CD94+NKG2)	239	239	239	239	239
Residues in Category	54	28	30	7	25
exact p	0.021	0.006	0.152	0.003	0.044
cumulative p	0.031	0.008	0.266	0.003	0.061
p “distribution by chance”	<0.05	<0.01	NS	<0.01	∼0.06

Binomial test to investigate whether positively selected residues are randomly distributed or are clustered at functionally relevant sites of the molecules. Several CD94/NKG2 functional categories were considered based on the human CD94/NKG2A crystal structure [22],[23]: residues contacting the peptide presented by the MHC ligand form the ‘Peptide’ category while those contacting the MHC class I heavy chain form the ‘MHC’ category; residues mediating heterodimer formation represent the ‘Dimer’ category. Additionally, Peptide and MHC contacts (‘Ligand binding’) and all known contact residues (‘All’) are summarised. Details on the residues in each category are in Figure S6.

To fully investigate the extent of allelic diversity of ‘lower’ primate *NKC* genes we specifically amplified the exons encoding the lectin-like domain of mouse lemur *CD94*, *NKG2*, and *Ly49L* genes in a cohort of 46 free-living unrelated animals derived from the Kirindy region in Madagascar [24]. Twelve animals were analysed for all genes and further 34 animals were additionally analysed for the three *CD94* genes. No presence/absence polymorphisms of grey mouse lemur *CD94* or *NKG2* genes were observed in our cohort, indicating that copy number variation similar to what is known for human *KIR* and mouse/rat *Ly49* haplotypes is not evident in grey mouse lemur *CD94*/*NKG2* haplotypes. Single nucleotide polymorphisms (SNPs) were detected for all genes and with the exception of *CD94-3* and *NKG2D*, all the *NKC* genes show an excess of non-synonymous substitutions (Table S1). While 30% (71/236) of all the codons forming the lectin-like domains of CD94 and NKG2 have non-synonymous allelic polymorphisms in at least one of the ‘lower’ primate *CD94* or *NKG2* genes, 66% (47/71) of these codons are neither sites involved in ligand binding or dimer formation nor positions we detected as positively selected in our gene analysis. Because the differences between the genes largely overshadowed allelic differences in our analysis for positive selection, such an observation suggests that following gene duplication natural selection first diversified functional sites and subsequently favoured in each species polymorphisms at sites often not directly involved in function, presumably to further ‘tweak’ functions.

**Figure 6:**
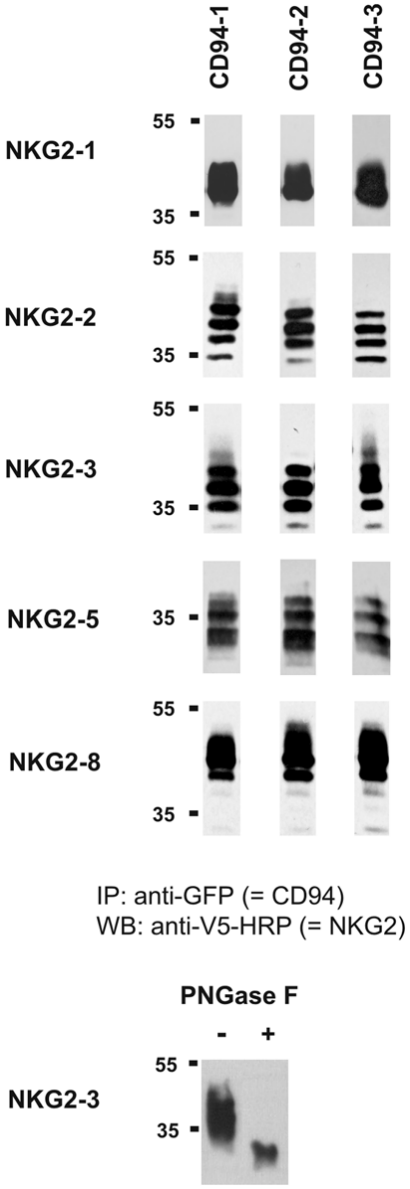
Co-immunoprecipitation of various combinations of grey mouse lemur CD94 and NKG2 expression constructs. CD94 constructs fused at the NH2 terminus with GFP and NKG2 molecules tagged at the COOH terminus with the V5 tag were transiently transfected into 293T cells. Analyses were performed 24h upon transfection. Representatives of at least three independent experiments are shown. Differential degree of glycosylation gives rise to multiple bands. In the lower panel deglycosylation with PNGase F is demonstrated exemplarily for inhibitory receptor NKG2-3.

**Figure 7:**
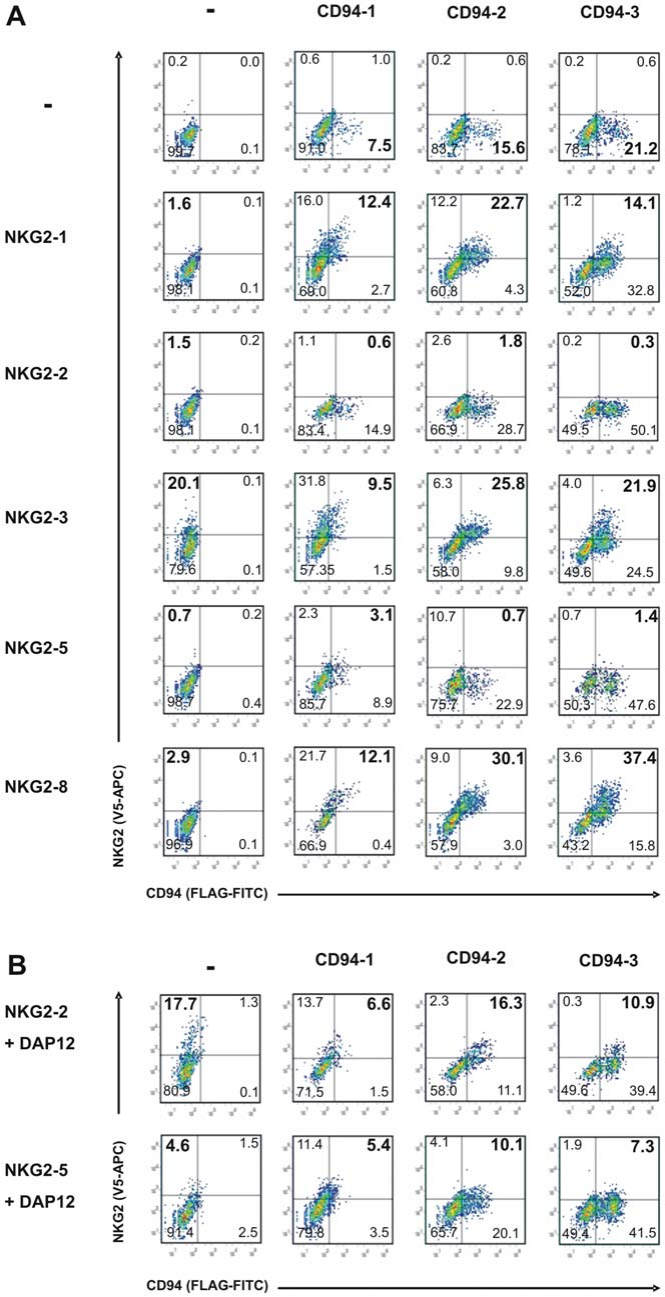
COOH terminally tagged NKG2 (V5 tag) and CD94 (FLAG tag) molecules were transiently transfected into 293T cells. Analyses were performed 24 h upon transfection. Representatives of at least three independent experiments are shown. Numbers indicate the percentage of cells in each quadrant. Cells in the upper right quadrant show V5- and FLAG-double-positive cells. (A) CD94 and NKG2 cell surface expression without co-expression of DAP12. (B) Enhancement of NKG2-2 and NKG2-5 expression at the cell surface by co-expression of myc-tagged DAP12.

Unlike the ligand binding sites, the positions involved in dimer formation are not significantly enriched in positively selected sites (Table 2, Figure S6). Because of this apparent lack of major diversification of the dimer formation function, we hypothesised that the three CD94 and five NKG2 molecules of the grey mouse lemur can be freely combined to form various CD94/NKG2 heterodimers. To test this hypothesis, we checked all possible combinations with (extracellular) V5-tagged NKG2 and (intracellular) GFP-tagged CD94 molecules. Expression constructs were transiently transfected into 293T cells and CD94/NKG2 heterodimer formation was assayed by co-immunoprecipitation using tag-specific monoclonal antibodies. Heterodimers were found for all CD94/NKG2 combinations by co-immunoprecipitation (Figure 6). Cell surface expression of all CD94/NKG2 receptor combinations was examined with (both extracellular) FLAG- and V5-tagged CD94 and NKG2 molecules, respectively. All combinations of NKG2-1, NKG2-3, and NKG2-8 with the three CD94 molecules were found on the cell surface (Figure 7A). In contrast, cell surface expression of both NKG2-2 and NKG2-5 receptors in combination with CD94 molecules was either low (CD94-1/NKG2-5) or lacking (Figure 7A), suggesting that these receptors depend on the DAP12 adaptor molecule. An expression construct of mouse lemur *DAP12* with a c-myc-tag was established and expression was controlled in parallel experiments by intracellular staining (data not shown). Co-transfection with c-myc-tagged mouse lemur DAP12 significantly increased cell surface expression of all combinations formed by CD94/NKG2-2 and CD94/NKG2-5 heterodimers (Figure 7B). This finding identifies NKG2-2 and NKG2-5 as classical activating receptors whose cell surface expression is dependent on interaction with DAP12. CD94 and NKG2 expression constructs were also transfected in 293T cells alone. Similar to human CD94, all three mouse lemur CD94 proteins can be found at the cell surface in the absence of any NKG2 molecule, but unlike human NKG2 molecules, also mouse lemur NKG2-2 (with DAP12) and NKG2-3, and to a lower extent NKG2-5 (with DAP12) and NKG2-8, are expressed at the cell surface in the absence of CD94 (Figure 7A and 7B).

Taken together, our data show that three CD94 molecules can be combined with five NKG2 molecules to form 15 different NK cell receptors that display significant diversity at sites of interaction with MHC class I ligands and their bound peptide. This indicates that besides gene amplification and positive diversifying selection combinatorial diversity is also a mechanism that significantly contributes to the increase of NK cell receptor diversity in ‘lower’ primates.

## Discussion

The polymorphic NK cell receptors of humans and mice are functionally similar but structurally unrelated. Therefore, we traced back NK cell receptors and MHC class I ligands to the base of the primate evolutionary tree by analysis of the *LRC*, *NKC*, and *MHC* genomic regions in primates distantly related to humans. We demonstrate that ‘lower’ primates deviate from ‘higher’ primates in the usage of polymorphic NK cell receptors. Except for the *KIR3DX1* gene, lemurs and possibly other ‘lower’ primates have neither functional nor highly polymorphic *KIR* genes like their relatives, the catarrhine (Old World monkeys, apes, and humans) and platyrrhine (New World monkeys) primates, nor do they show an expansion of *Ly49* genes as in rodents. Instead, lemurs have considerably amplified and diversified their C-type lectin-like *CD94* and *NKG2* genes. We conclude from our findings that the NK cell receptor repertoire of ‘lower’ primates is at least as diverse as in ‘higher’ primates or rodents. Thus, in addition to the *KIR* and *Ly49* genes of ‘higher’ primates and rodents, the CD94/NKG2 heterodimers of lemurs represent a third system of polymorphic and diverse NK cell receptors. Compatible with such a system is that the duplicated lemur *CD94* and *NKG2* genes show sequence diversifications and strong signs of positive diversifying selection. In accordance with these characteristics, lemur *NKG2* genes do not show signs of gene homogenisation as opposed to ‘higher’ primate *NKG2* sequences [11]. Such homogenisation may serve to keep NKG2 amino acid sequences conserved for interaction with the invariable MHC-E ligand, a situation that is not observed for the polymorphic CD94/NKG2 receptors in ‘lower’ primates.

*CD94* gene duplications are not restricted to ‘lower’ primates from Madagascar as they were found in an African strepsirrhine primate (*Perodicticus potto*) and in the Asian tarsier (*Tarsius syrichta*). The latter is particularly interesting, as the tarsier is more closely related to ‘higher’ primates than to ‘lower’ primates [21]. Analysis of repetitive elements in *CD94* intron 4 sequences revealed that duplications had occurred repeatedly and independently in ‘lower’ primates. Thus, the polymorphic CD94/NKG2 system is likely present in many if not all ‘lower’ primates. This is in sharp contrast to the situation in ‘higher’ primates where *CD94* is a single copy, non-polymorphic and highly conserved gene.

**Figure 8:**
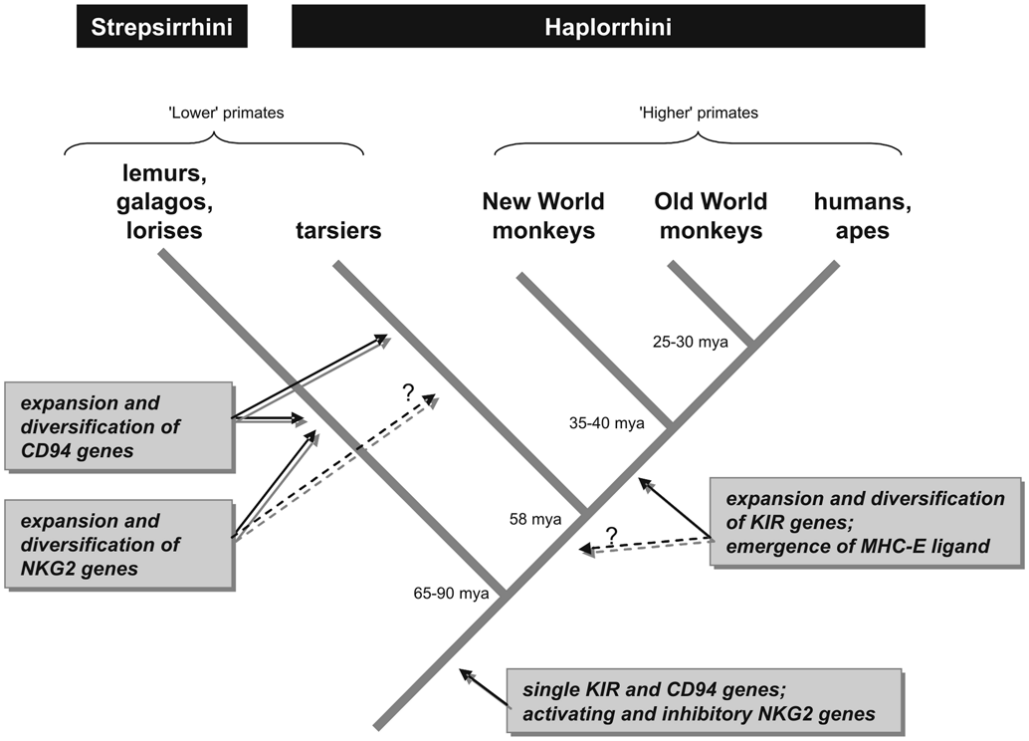
Model of NK cell receptor evolution in primates.

Three *KIR* genes were detected in the lemur *LRC* region, a functional and a pseudogene copy of *KIR3DX1* and a *KIR3DP* pseudogene. According to its characteristics, the *KIR3DP* gene may represent the ‘Ur-KIR’ gene of all ‘higher’ primate *KIRs*. This gene already contains the repetitive elements *MLTD1*, *MER70B*, *MSTB1* in its introns, which are assumed to be integrated about 60–100 mya [25], a time that is compatible with the splitting of lemurs and human of about 65–90 mya [26]. Thus, we postulate that KIR and CD94/NKG2 receptors evolved differently in primates: while in the lineage leading to ‘lower’ primates *CD94* and *NKG2* but not *KIR* genes expanded, the opposite happened in the lineage leading to ‘higher’ primates where *KIR* genes expanded and CD94 and NKG2 co-evolved with the non-classical MHC-E molecule to become a conserved receptor/ligand system (Figure 8). The finding that ‘lower’ primates did not amplify and diversify *KIR* or *Ly49* genes but, instead, evolved a polymorphic *CD94*/*NKG2* system, strengthens previous assumptions that mammals only utilise a single class of polymorphic NK cell receptors, despite their obvious ability to develop multiple classes [27],[28]. Most likely, this development is influenced by the pathogenic threat these organisms are subjected to and can involve different receptor types such as monomeric KIR, heterodimeric CD94/NKG2, and homodimeric Ly49. Nevertheless, mammalian species are equipped with all types of receptor genes [1], which gives some flexibility for adaptation according to NK cell receptor and ligand requirements.

All *class I* genes in the *MHC* equivalent genomic region on grey mouse lemur chromosome 6 are pseudogenes and all functional *MHC class I* genes were translocated to chromosome 26. Additionally, no functional *MIC* gene could be identified in any of the *class I* gene-containing regions in the grey mouse lemur. However, this finding is not surprising, as NKG2D ligands are numerous and functionally redundant [29], absence of *MIC* is evident in rodents [30] and deletions of both *MICA* and *MICB* were reported in East-Asians [31]. In addition to this unusual organisation of the grey mouse lemur *MHC*, a striking difference to ‘higher’ primates is the apparent absence of a strict *HLA-E* orthologue or functional homologue. As the BAC library was screened exhaustively and in the light of our genome-wide approach published earlier [18], it appears rather unlikely that *MHC-E*-like class I genes were not detected. Thus, the putative CD94/NKG2 ligands are expected among the sequenced *MHC class I* genes. Nevertheless, we cannot completely rule out the possibility that a *HLA-E*-like gene was not detected by our approach or that a gene with HLA-E-like function is among the detected *MHC class I* genes. There has been some debate in the past on whether HLA-E and mouse H2-Qa1 have a common origin [32] or whether their similarity in the peptide-binding region is a consequence of convergent evolution [33]. In the light of our data, the second hypothesis appears more likely, as diversified KIR and conserved MHC-E/CD94/NKG2 emerged only in ‘higher’ primates and diversified Ly49 and conserved H2-Qa1/CD94/NKG2 evolved independently in rodents.

The observed positive diversifying selection in ‘lower’ primates is more pronounced in the three *CD94* than the *NKG2* genes, suggesting that the CD94 molecules have significant impact on the binding of the polymorphic MHC class I ligands and their bound peptides. Indeed, the recently determined three-dimensional structure of human CD94/NKG2A in complex with HLA-E revealed that CD94 and NKG2A contribute about 80% and 20%, respectively, to the interaction with HLA-E and its bound peptide [22],[23]. Translated to the situation in ‘lower’ primates, duplication of *CD94* genes and their strong sequence diversification by positive diversifying selection reflects the requirement to maintain binding to polymorphic (classical) MHC class I molecules and bound peptides. Finally, we demonstrate that all possible CD94/NKG2 combinations are able to form heterodimers at the cell surface, revealing important implications: exchange of the CD94 or the NKG2 subunit is likely to influence the binding specificity for MHC class I ligands and their bound peptide and the functional properties (inhibitory or stimulating) of the receptor. For example, the combination of the three CD94 and five NKG2 molecules in the grey mouse lemur or three CD94 and eight NKG2 molecules in the ruffed lemur gives rise to 15 or 24 different NK cell receptors, respectively. Thus, we conclude that the NK cell receptor repertoire in ‘lower’ primates is not mainly achieved by duplications, but rather by combinatorial diversity, a phenomenon that was so far unknown for any NK cell receptor. NK cells were recently shown to exhibit features of adaptive immunity, namely immunological memory [34]. Combinatorial diversity of immune receptors is a further hallmark of adaptive immunity. Although the combinatorial diversity of ‘lower’ primate CD94/NKG2 receptors is obviously much less than that usually seen for B or T cell receptors, our data additionally highlight the close relationship of two lymphocyte subsets: natural killer cells and cytotoxic T cells.

In summary, we have uncovered a ‘third way’ of polymorphic and diversified NK cell receptors in mammals. The CD94/NKG2 receptor system (and not *KIR*) of ‘lower’ primates is characterised by duplication, sequence diversification by means of positive diversifying selection and allelic diversity. Consistent with this highly dynamic CD94/NKG2 system, the MHC class I molecules as putative ligands of these receptors show strong signs of co-evolution and an unusual chromosomal organisation. CD94 and NKG2 subunits constitute the main NK cell receptor repertoire in ‘lower’ primates and are freely combinable. This finding discloses a so far unknown mechanism of generating the NK cell receptor diversity: combinatorial diversity.

## Materials and Methods

### Ethics statement

All experiments were carried out in accordance with the French Rural Code Directive (articles R21-87-90), the German Animal Welfare Law, guidelines of the German Research Foundation, and the European Communities Council Directive (86/609/EEC). Field sampling of *Microcebus murinus* tissue samples in Madagascar was conducted under the autorisation of the Ministère de l''Environnement, des Forêts et du Tourisme of the Republic of Madagascar. Samples were exported under a CITES permit by the Bundesamt für Naturschutz, Bonn, Germany.

### Blood and DNA samples

Grey mouse lemurs (*Microcebus murinus*) and black-and-white ruffed lemurs (*Varecia variegata*) are housed in the facilities of the UMR CNRS/MNHN and the German Primate Center, respectively. Blood was obtained during regular veterinary inspections. DNA samples from potto and tarsier were kindly obtained from Helga Schulze (University of Bochum, Germany) and Jürgen Schmitz (University of Münster, Germany).

### BAC library screening and clone sequencing

Filters of BAC library CHORI-257 derived from a female grey mouse lemur (*Microcebus murinus*) were obtained from BACPAC Resources at the Children's Hospital Oakland Research Institute (http://bacpac.chori.org/home.htm). Filters were screened with gene probes for human *KIR2DL4*, *LILRA2*, *NCR1*, and rhesus macaque *KIR3DX1* (*LRC* region), human *CD94*, *NKG2A*, and *Ly49L* (*NKC* region), catta *BAT1*, *POU5F1*, *TCF19*, and rat *Gnl1*, *Cat56*, *Trim39*, *Trim26*, *Trim10*, *Trim15*, *Ppp1r11*, *Mog*, and mouse lemur *MHC class I* (*MHC* class I region). Screening with radioactively labelled probes was done according to the supplier's recommendations.

BAC clone DNA was purified by CsCl density centrifugation and isolated DNA was sheared by sonification. DNA fragments of 1.5–3.5 kb were selected and cloned into plasmid pUC19. Inserts were amplified by PCR using insert-flanking M13 forward and reverse primers and amplificates were sequenced with Applied Biosystems BigDye terminator chemistry and analyzed in ABI3730xl sequencers (Applied Biosystems). Raw sequences were processed by Phred (www.phrap.org) and assembled into a contiguous sequence by Phrap (www.phrap.org). Both programs are available from Phil Green, University of Washington. Exons and introns of genes were identified in finished BAC clone sequences manually or by BLAST and FGENESH-2 algorithms (http://www.ncbi.nlm.nih.gov/BLAST/; http://www.softberry.com/all.htm). All BAC clone sequences have ‘finished sequencing’ quality (1 putative mistake/100,000 bases) and the data have Phred values of 60.

Exons 4 to 6 of the grey mouse lemur *CD94*, *NKG2*, and *Ly49L* genes were amplified from genomic DNA of 12 (all genes) and additional 34 (only *CD94* genes) unrelated free-living grey mouse lemur individuals from the Kirindy region in Madagascar. Specific primers (Table S2) were designed to be located in exon-flanking introns. PCR products were completely sequenced on both strands and SNPs were identified.

Primers were designed for amplification of conserved regions of potto and tarsier *CD94* exon 4, intron 4 and exon 5 (Table S2). Genomic DNA was used of a single potto (*Perodicticus potto*) and a single Philippine tarsier (*Tarsius syrichta*) individual. PCR products were cloned and sequenced. For every *CD94* sequence at least two identical clones were identified.

DDBJ/EMBL/GenBank database accession numbers for BAC clones of *MHC class I* regions (AB480748, FP236831, FP236832, FP236833, FP236839), *NKC* region (haplotype 1: FP236838, haplotype 2: FP236834), *LRC* region (CR974412, CR974436, CR974413), and cDNA of grey mouse lemur and ruffed lemur *CD94*, *NKG2* and *Ly49L* sequences as well as potto and tarsier *CD94* sequences were assigned accession numbers FJ869057-FJ869114. Grey mouse lemur *KIR3DX1* and *DAP12* cDNA sequences are found under FJ882074-FJ882079.

### RT–PCR and expression constructs

Total RNA was extracted from a liver sample of a grey mouse lemur and a blood sample from a further grey mouse lemur housed at UMR CNRS/MNHN in Brunoy (France) and a blood sample from a black-and-white ruffed lemur (*Varecia variegata variegata*) housed at the German Primate Center. Reverse transcription was performed with M-MLV reverse transcriptase (Promega). Based on the mouse lemur BAC sequences primers were designed to obtain the complete open reading frames of mouse lemur and ruffed lemur *NKC* genes. cDNA sequences of the ruffed lemur were completed by 5′- and 3′-RACE PCR with the GeneRacer Kit (Invitrogen). All cDNA sequences were cloned and sequenced. For interaction studies, grey mouse lemur *CD94* and *NKG2* cDNA sequences were inserted into the pcDNA3.1/NT-GFP-TOPO and the pcDNA3.1/V5-His-TOPO vector (Invitrogen), respectively. For cell surface expression, the three *CD94* cDNA sequences were expressed without GFP at the NH_2_ terminus, but with a FLAG tag (DYKDDDDK) at the COOH terminus for extracellular detection. The grey mouse lemur *DAP12* cDNA was isolated by RT-PCR. The encoded DAP12 was fused at the COOH terminus with the c-myc peptide epitope (EQKLISEEDL). Primer sequences and performed PCRs are listed in Table S2.

### Transfection, co-immunoprecipitation, and flow cytometry

CD94 and NKG2 molecules were tagged with GFP and with V5 for co-immunoprecipitation experiments. To avoid any potential unwanted interactions between the tags, GFP was fused to the amino terminus of CD94 ( =  intracellular localisation) and V5 to the carboxy terminus of NKG2 molecules ( =  extracellular localisation). Expression constructs were transiently transfected in the human 293T cell line using Metafectene (Biontex GmbH). 24 h after transfection, cells (1×10^7^ cells/ml) were lysed in NP-40 lysis buffer containing 0.1% NP-40, 50 mM Tris, pH 7.6, 150 mM NaCl, 4 mM EDTA and protease inhibitors (Roche). Lysates were incubated overnight at 4°C with monoclonal mouse anti-GFP antibody (Clontech) and then incubated with protein G Sepharose 4 Fast Flow beads (GE Healthcare) for 3 h. Sepharose beads were washed five times with lysis buffer and bound proteins were eluted with loading buffer at 95°C for 10 minutes. Samples were electrophoresed in 10% polyacrylacrylamide gels and transferred to nitrocellulose membrane. Western blotting was performed with HRP-coupled monoclonal mouse anti-V5 antibody (Invitrogen) to test for co-immunoprecipitated V5-tagged NKG2 molecules. Deglycosylation was performed with PNGase F according to supplier's recommendations (New England Biolabs).

For analysis of CD94/NKG2 receptors on the cell surface, CD94 was FLAG-tagged at the carboxy terminus ( =  extracellular localisation). Respective expression constructs of CD94 together with the V5-tag NKG2 constructs (see above) were transiently transfected in 293T cells. Cell surface expression of CD94/NKG2-2 and CD94/NKG2-5 receptors were tested by additional transient transfection with c-myc-tagged mouse lemur DAP12 expression constructs. As control, single transfections with either CD94 or NKG2 expression constructs were performed. Potential formation of heterodimers of grey mouse lemur NKG2 and human CD94 (from 293T cells) was excluded by testing with an anti-human CD94 antibody (AbD Serotec). 24 hours post transfection cells were detached from cell culture dishes and washed twice with 1× PBS. Cells were stained with APC-labelled anti-V5 (Abcam) and FITC-labelled anti-FLAG (Sigma-Aldrich) monoclonal antibodies for surface expression of NKG2 and CD94. In parallel experiments, expression of DAP12 was monitored with an anti-c-myc mouse monoclonal antibody (Sigma-Aldrich) by intracellular staining of cells, which were previously fixed with 1.5% paraformaldehyde (Merck) and permeabilized with 0.25% saponin (Roth), and binding was detected by a PE-Cy5-labelled goat anti-mouse IgG polyclonal antibody (Santa Cruz Biotechnology). After washing twice with 1× PBS cells were resuspended in 200 µl of 1× PBS and 50,000 events were measured. Living cells were gated based on forward and side scatter characteristics and analysed for APC and FITC staining. All samples were analysed in a LSRII flow cytometer (BD). Data were acquired with BD FACS Diva 5.1 software (BD) before analysis with FlowJo 7.2.9 software (TreeStar).

### Phylogenetic analyses

*CD94*, *NKG2* and *MHC class I* nucleotide sequences were aligned with MAFFT [35] and corrected manually. Phylogenetic analyses were conducted using three methods: neighbor-joining (NJ), parsimony and Bayesian phylogenetics. NJ analyses were performed with MEGA3.1 [36] using the Tamura-Nei method with 1,000 replicates. PAUP*4.0b10 [37] and the tree bisection-reconnection branch swapping algorithm were used for parsimony analyses with 1,000 replicates and a heuristic search. For the Bayesian analysis, the model of DNA substitution was selected using MODELTEST3.7 [38] and the Akaike information criterion. Bayesian phylogenetic analyses were conducted with MRBAYES3.1.2 [39]; sampling was performed with one cold chain and three heated chains, which were run for 10^6^ generations or until average standard deviation of split frequencies was <0.01. Trees were sampled every 200 generations and the first 2,500 trees were discarded before a consensus tree was generated; three simultaneous runs were conducted and average standard deviation of split frequencies was always <0.01. The tree topologies obtained with the three methods were compared with PAUP*4.0b10 using the Shimodaira-Hasegawa test of alternative phylogenetic hypotheses with re-sampling estimated log-likelihood optimization, and 10,000 bootstrap replicates; in all analyses the test failed to reject any of the alternative tree topologies (α = 0.05). This comparison was made with the maximum likelihood model defined by MODELTEST. A maximum-likelihood analysis was also performed for the study of the NKG2 cytoplasmic and transmembrane sequences using RAxML7 [40] under the GTR+CAT model with 1,000 replicates (rapid bootstrapping).

### Selection analyses

dN/dS (ω) ratios for CD94 and NKG2 lectin-like domains were estimated using PAML v3.15 [41] with the F3 X 4 model of codon frequencies. Bayesian tree topologies were used for these analyses and three sets of likelihood ratio tests were conducted to compare null models that do not allow ω>1 (M1a, M7 and M8a) with models that do (M2a and M8). Significance was assessed by comparing twice the difference in likelihood between the models (2ΔL) to a χ2 distribution with one (M8a/M8) or two (M1a/M2 and M7/M8) degrees of freedom. Codons with ω>1 were identified using the Bayes Empirical Bayes approach [42].

The distribution of the selected sites in the CD94/NKG2 lectin-like domains was studied using a binomial distribution: considering Ω = (0,1,2,…,n), ∀k ∈ Ω, p = (X = k) =  _n_C_k_ * p^k^ * q^n−k^. This indicates for example that under a random distribution it is unlikely to have more than 5 of the 21 positively selected sites in the ligand binding region that represents 28 of the 239 total sites (α = 0.05); so the fact that we observe 7 of the 21 positively selected sites in this region indicates a distribution biased toward this region (α  = 0.01).

## Supporting Information

Figure S1 Mouse lemur *KIR3DX1* sequence. (A) cDNA, (B) deduced amino acid sequence and (C) genomic sequence. cDNA sequence was determined by RT-PCR of mouse lemur PBMC. Start codon is marked in green, stop codon in red, exons in grey, and ITIM in the protein sequence in yellow. Nucleotides and corresponding amino acid residues that are absent in an alternatively spliced product are shown in italics. Transmembrane region is underlined.
(0.04 MB PDF)

Figure S2 Multiple sequence alignments of CD94 proteins. (A) Multiple sequence alignment of mouse lemur, ruffed lemur, human, and common marmoset CD94 proteins. Sequences are subdivided into cytoplasmic (CY), transmembrane (TM), stalk, and C-type lectin-like domain (CTLD). (B) Amino acid sequence identities (in %) of mouse lemur, ruffed lemur, human and common marmoset CD94. Mimu, *Microcebus murinus*; Vava, *Varecia variegata*; Hosa, *Homo sapiens*; Caja, *Callithrix jacchus*.
(0.01 MB PDF)

Figure S3 Multiple sequence alignments of NKG2 proteins. (A) mouse lemur and (B) ruffed lemur NKG2 and Ly49L amino acid sequences. Sequences are subdivided into cytoplasmic (CY), transmembrane (TM), stalk, and C-type lectin-like domain (CTLD). ITIM are highlighted in green, YxxM motif in yellow and positively charged amino acids in the TM in red. An ITIM at unusual position was found in mouse lemur NKG2-5 and is marked in blue. It is not clear whether this motif functions as an ITIM. Due to failure of obtaining a 5′-RACE product for ruffed lemur *Ly49L*, the corresponding deduced amino acid sequence is incomplete at the amino terminal end. The two identified ruffed lemur Ly49L alleles show only a single synonymous substitution and have therefore identical deduced amino acid sequences. Mimu, *Microcebus murinus*; Vava, *Varecia variegata*.
(0.02 MB PDF)

Figure S4 *CD94* exon 4 to exon 5 sequence alignment of (A) potto (*Perodicticus potto*) and (B) Philippine tarsier (*Tarsius syrichta*). The shading threshold is 55% sequence identity. N in the sequences of potto *CD94-6* and *-7* and of tarsier *CD94-9* denotes an unknown number of thymidine nucleotides. The *CD94-7* sequence of the tarsier contains a stop codon in exon 4 which is highlighted in bold and underlined. Pepo, *Perodicticus potto*; Tasy, *Tarsius syrichta*. Alu elements are underlined.
(0.03 MB PDF)

Figure S5 Phylogenetic analyses of MHC class I genes with complete coding sequences excluding the peptide binding residues. Analysis and display are as described for Figure 3. Numbers as gene names for MHC class I are the same as in Figure 1C. Non-primate sequences included are mouse (H2) and rat (RT1). Peptide binding residues were defined according to Bjorkman et al. [44]. Patr, *Pan troglodytes*; Gogo, *Gorilla gorilla*; Hyla, *Hylobates lar*; Saoe, *Saguinus oedipus*; Sasc, *Saimiri sciureus*; Mamu, *Macaca mulatta*; Mimu, *Microcebus murinus*; Aotr, *Aotus trivirgatus*; Caja, *Callithrix jacchus*; Pipi, *Pithecia pithecia*.
(0.07 MB PDF)

Figure S6 Contacts of CD94/NKG2 to bound peptide (Peptide) and to the MHC class I heavy chain (MHC) as well as residues mediating heterodimer formation (Dimer) based on the human CD94/NKG2A crystal structure [22],[23]. Additionally, Peptide and MHC contacts (Ligand binding) and all known contact residues (All) are summarised.
(4.54 MB TIF)

Table S1 Nucleotide and amino acid substitutions between different alleles of mouse lemur *CD94*, *NKG2*, and *Ly49L* sequences. SNPs were determined by sequencing the exons coding for the lectin-like domain (exon 4–6) of 12 individuals for *NKG2* and *Ly49L*. For *CD94* a further 34 animals were analysed.
(0.04 MB PDF)

Table S2 Primer sequences and performed PCRs.
(0.08 MB PDF)

## References

[1] Kelley J, Walter L, Trowsdale J (2005). Comparative genomics of natural killer cell receptor gene clusters.. PLoS Genet.

[2] Lanier LL (2005). NK cell recognition.. Annual review of immunology.

[3] Kärre K (2002). NK cells, MHC class I molecules and the missing self.. Scandinavian journal of immunology.

[4] Moretta L, Bottino C, Pende D, Vitale M, Mingari MC (2005). Human natural killer cells: Molecular mechanisms controlling NK cell activation and tumor cell lysis.. Immunol Lett.

[5] Uhrberg M, Valiante NM, Shum BP, Shilling HG, Lienert-Weidenbach K (1997). Human diversity in killer cell inhibitory receptor genes.. Immunity.

[6] Cadavid LF, Lun CM (2008). Lineage-specific diversification of killer cell Ig-like receptors in the owl monkey, a New World primate.. Immunogenetics.

[7] Sambrook JG, Bashirova A, Palmer S, Sims S, Trowsdale J (2005). Single haplotype analysis demonstrates rapid evolution of the killer immunoglobulin-like receptor (KIR) loci in primates.. Genome research.

[8] Nylenna O, Naper C, Vaage JT, Woon PY, Gauguier D (2005). The genes and gene organization of the Ly49 region of the rat natural killer cell gene complex.. Eur J Immunol.

[9] Uhrberg M (2005). The KIR gene family: life in the fast lane of evolution.. Eur J Immunol.

[10] Parham P (2005). MHC class I molecules and KIRs in human history, health and survival.. Nat Rev Immunol.

[11] Averdam A, Kuhl H, Sontag M, Becker T, Hughes AL (2007). Genomics and diversity of the common marmoset monkey NK complex.. J Immunol.

[12] Yokoyama WM, Plougastel BF (2003). Immune functions encoded by the natural killer gene complex.. Nat Rev Immunol.

[13] Braud VM, Allan DS, O'Callaghan CA, Soderstrom K, D'Andrea A (1998). HLA-E binds to natural killer cell receptors CD94/NKG2A, B and C.. Nature.

[14] Vance RE, Kraft JR, Altman JD, Jensen PE, Raulet DH (1998). Mouse CD94/NKG2A is a natural killer cell receptor for the nonclassical major histocompatibility complex (MHC) class I molecule Qa-1(b).. The Journal of experimental medicine.

[15] Braud V, Jones EY, McMichael A (1997). The human major histocompatibility complex class Ib molecule HLA-E binds signal sequence-derived peptides with primary anchor residues at positions 2 and 9.. European journal of immunology.

[16] Guethlein LA, Older Aguilar AM, Abi-Rached L, Parham P (2007). Evolution of Killer Cell Ig-Like Receptor (KIR) Genes: Definition of an Orangutan KIR Haplotype Reveals Expansion of Lineage III KIR Associated with the Emergence of MHC-C.. J Immunol.

[17] Sambrook JG, Bashirova A, Andersen H, Piatak M, Vernikos GS (2006). Identification of the ancestral killer immunoglobulin-like receptor gene in primates.. BMC genomics [electronic resource].

[18] Flügge P, Zimmermann E, Hughes AL, Günther E, Walter L (2002). Characterization and phylogenetic relationship of prosimian MHC class I genes.. J Mol Evol.

[19] Roos C, Schmitz J, Zischler H (2004). Primate jumping genes elucidate strepsirrhine phylogeny.. Proceedings of the National Academy of Sciences of the United States of America.

[20] Vilches C, Parham P (2002). KIR: diverse, rapidly evolving receptors of innate and adaptive immunity.. Annual review of immunology.

[21] Schmitz J, Roos C, Zischler H (2005). Primate phylogeny: molecular evidence from retroposons.. Cytogenetic and genome research.

[22] Kaiser BK, Pizarro JC, Kerns J, Strong RK (2008). Structural basis for NKG2A/CD94 recognition of HLA-E.. Proc Natl Acad Sci USA.

[23] Petrie EJ, Clements CS, Lin J, Sullivan LC, Johnson D (2008). CD94-NKG2A recognition of human leukocyte antigen (HLA)-E bound to an HLA class I leader sequence.. The Journal of experimental medicine.

[24] Eberle M, Kappeler PM (2004). Selected polyandry: female choice and inter-sexual conflict in a small nocturnal solitary primate (Microcebus murinus).. Behav Ecol Sociobiol.

[25] Martin AM, Freitas EM, Witt CS, Christiansen FT (2000). The genomic organization and evolution of the natural killer immunoglobulin-like receptor (KIR) gene cluster.. Immunogenetics.

[26] Steiper ME, Young NM (2006). Primate molecular divergence dates.. Mol Phyl Evol.

[27] Parham P (2008). The genetic and evolutionary balances in human NK cell receptor diversity.. Seminars in immunology.

[28] Sambrook JG, Beck S (2007). Evolutionary vignettes of natural killer cell receptors.. Current opinion in immunology.

[29] Eagle RA, Trowsdale J (2007). Promiscuity and the single receptor: NKG2D.. Nat Rev Immunol.

[30] Hurt P, Walter L, Sudbrak R, Klages S, Müller I (2004). The genomic sequence and comparative analysis of the rat major histocompatibility complex.. Genome Res.

[31] Komatsu-Wakui M, Tokunaga K, Ishikawa Y, Leelayuwat C, Kashiwase K (2001). Wide distribution of the MICA-MICB null haplotype in East Asians.. Tissue antigens.

[32] Joly E, Rouillon V (2006). The orthology of HLA-E and H2-Qa1 is hidden by their concerted evolution with other MHC class I molecules.. Biology direct.

[33] Yeager M, Kumar S, Hughes AL (1997). Sequence convergence in the peptide-binding region of primate and rodent MHC class Ib molecules.. Molecular biology and evolution.

[34] Sun JC, Beilke JN, Lanier LL (2009). Adaptive immune features of natural killer cells.. Nature.

[35] Katoh K, Misawa K, Kuma K, Miyata T (2002). MAFFT: a novel method for rapid multiple sequence alignment based on fast Fourier transform.. Nucleic acids research.

[36] Kumar S, Tamura K, Nei M (2004). MEGA3: Integrated software for Molecular Evolutionary Genetics Analysis and sequence alignment.. Brief Bioinform.

[37] Swofford DL (2001). PAUP*: Phylogenetic analysis using parsimony (*and other methods), version 4.0., Sinauer, Sunderland, Massachusetts.

[38] Posada D, Crandall KA (1998). MODELTEST: testing the model of DNA substitution.. Bioinformatics (Oxford, England).

[39] Ronquist F, Huelsenbeck JP (2003). MrBayes 3: Bayesian phylogenetic inference under mixed models.. Bioinformatics (Oxford, England).

[40] Stamatakis A (2006). RAxML-VI-HPC: maximum likelihood-based phylogenetic analyses with thousands of taxa and mixed models.. Bioinformatics (Oxford, England).

[41] Yang Z (1997). PAML: a program package for phylogenetic analysis by maximum likelihood.. Comput Appl Biosci.

[42] Yang Z, Wong WS, Nielsen R (2005). Bayes empirical bayes inference of amino acid sites under positive selection.. Molecular biology and evolution.

[43] Dutrillaux B (1979). Chromosomal evolution in primates: Tentative phylogeny from Microcebus murinus (Prosimian) to man.. Hum Genet.

[44] Bjorkman PJ, Saper MA, Samraoui B, Bennett WS, Strominger JL (1987). Structure of the human class I histocompatibility antigen, HLA-A2.. Nature.

[45] DeLano WL (2002). The PyMOL Molecular Graphics System.

