# Supplementary material for: A Novel System of Polymorphic and Diverse NK Cell Receptors in Primates
Source: PLoS Genet. 2009 Oct 16;5(10):e1000688. doi: 10.1371/journal.pgen.1000688 (PMC2757895; doi:10.1371/journal.pgen.1000688)
Supplement: Figure S1 — Mouse lemur KIR3DX1 sequence. (A) cDNA, (B) deduced amino acid sequence and (C) genomic sequence. cDNA sequence was determined by RT-PCR of mouse lemur PBMC. Start codon is marked in green, stop codon in red, exons in grey, and ITIM in the protein sequence in yellow. Nucleotides and corresponding amino acid residues that are absent in an alternatively spliced product are shown in italics. Transmembrane region is underlined. (0.04 MB PDF) [file pgen.1000688.s001.pdf]

A *KIR3DX1* cDNA sequence

ATGTCCTCCAATCTCATGCCTCTCCTATGTCTAGGATTCTACCTAGGCCACAGGACCTTCACACACATGG*GTG*
GTCACAACCAGCCTTCCCTGTCAGCCTCGCCGAGCCCTGTGGTCCCCCTAGGAGGACATGTGACACTTCGCTG
*TTATTCCCACCTTTCATTTGTCATAG*TCAAACTATCCAAAAGAGTTGGGAGCAACATCTCTGAGATCTACAGT
GTCCTTTCTCACAACACCTTTGCCTTCAACCCGGTCACCACAGCACATGCAGGGACCTACAGATGTTCTGTAT
CTTACACCCAGAATCCCTCTGTGTGGTCAGCACTCAGCGAACCCCTGGAGATCATGGTCACAGGTGTGTTCAG
GAAACCCTCCATCTCAGCATTCCCAAGCTCCTTAGTGCACACAGGAAGCAGTGTGACCCTGAGCTGTCTCTCA
GAACTGGACTTTGATAAATTTATCCTGCACAAGGTTGGGACCACACAGCATTCCCAGCTGCCTGCCAAGAGGG
TCCATGCTGAGCTCCCCTACACCCAGGCTGACTTCTCCATAGATACCATGATGCCCACCCATGCAGGGACCTA
CAGATGCTACGGTTCTCTCAGTCACTCTCCCTGTCAGTGGTCAGCTCCCAGTGACCCCCTGGACGTTGTGATC
ACCGGACAATATGAAAAGCCTTCTCTCTCCACCCATGTGGGCCCCACGATGGGCTCAGGAGAGAACATGACCT
TGTCCTGCAGCTCTAAGACCCGATTTGACAAGTACCATCTGTCCAGGGAGGGGGAGGCCCATGACCGCTGGCT
CAGTGAACGCCAGAGCCACAATGGAACATTCCAAGTCTACCTACCTCTGGGCTCTGAAACCCCTTCCCACGGA
GGGACCTATAGATGCTACGGCTACTTCTATTTCTCCCCCCATAAGTGGTCATCCCCAAGTGACCCCCTGCACC
TTTCAGTCACAGGAACCCCTAAGAGTACTTGTCTATCACCCATAGAATCAACCTCCAAATCCAAAGCAGGTCC
ACCTCCAGGACAGTTCCATAAACAGGACATTCTCATTGGACTCTCAGGAGTCATCATCTCCATTGGCATTTTC
CTCTCTGTTCTTCTTGGTTACTGGTGTTCTACCAAAAATCATCCTGCCATCATGGACACAGAGCCCATGGAAG
TCCAAATAATGGAGAGAAATGACCCTGCAGCAGAAGAAACACAGGAGATAGTATATGCTCAGCTAAACCAGCA
CGCACTCACACGGAGAGAATTCAGTCTCACTTCCCAGTGTTCCAGATACCGCTTGGATGAGCCCAGTACCTGG
GTGGACCTCCACACTTGCCACGTGGACCACGCTGAGGCCAGACCTGACCCTGGTCTCTGA

B KIR3DX1 amino acid sequence

MSSNLMPLLCLGFYLGHRTFTHM*GGHNQPSLSASPSPVVPLGGHVTLRCYSHLSFVI*VKL
SKRVGSNISEIYSVLSHNTFAFNPVTTAHAGTYRCSVSYTQNPSVWSALSEPLEIMVTGV
FRKPSISAFPSSLVHTGSSVTLSCLSELDFDKFILHKVGTTQHSQLPAKRVHAELPYTQA
DFSIDTMMPTHAGTYRCYGSLSHSPCQWSAPSDPLDVVITGQYEKPSLSTHVGPTMGSGE
NMTLSCSSKTRFDKYHLSREGEAHDRWLSERQSHNGTFQVYLPLGSETPSHGGTYRCYGY
FYFSPHKWSSPSDPLHLSVTGTPKSTCLSPIESTSKSKAGPPPGQFHKQDILIGLSGVII
SIGIFLSVLLGYWCSTKNHPAIMDTEPMEVQIMERNDPAAEETQEIVYAQLNQHALTRRE
FSLTSQCSRYRLDEPSTWVDLHTCHVDHAEARPDPGL*

C *KIR3DX1* genomic sequence

ATGTCCTCCAATCTCATGCCTCTCCTATGTCTAGgtgagtcctggaacgggagggaggagggtattaggatgg
aaaatgatgtcctgaagacctgggctctccacatcccagggctggaggtggaaatccataggaagccaagttc
atccatcctctgagcccaggctgagctaccaagtctgatatccatgacaaggcccagttatagggagggcagg
attcatttgggacaagtcatctctgatgtggtttttcctgcagGATTCTACCTAGGCCACAGGACCTTCACA
CACATGGgtgagtcccctgaattctcaggatgcagtggtggctaaacacaaagggatcccccaggtggaaaga
atggagcatggggtcgagttgacacaggtgatgttggaagggctttgtagtgattctccttggggcatagtag
tgaagtcccctggagaccctggaccctcatttgtcctggagaccctccatagacacccccctctaggaacagc
ctgttcctggaggagcctgtaatgtttagaactcggggtatctggggatgcactgaaatgatctctgtcagac
gggaaggactccatgctgggctccatggaactctttgagcaccaggagtaacgcagtttctcctgcccgcctg
tgagtctgagctaaagggcttcctgctgccagggtctgaggagggacccatcagcaggcagtgacctcatgga
cataggagcacattcctggggaggagaactattgcagtgagtttcacaagttgctttctatggcttgaatcca
gtagatttcccctttatgacatgtaggtattgttactgtaatagagatatttaagagcaaccttgtcgagcat
tttatcaacactctaagtaaatcttgtcctgggaagacctgccttgtccatgctggggtgcactgtgcccctg
agccaccagcgggtaccccctgggtgtcagagggctccagtttggggtaaaatccttggaagaatgtgaaggag
gaaaagcccccacaggacagtgggggcagggggcggctccacatttcttccctaagacttgcctcgtcctgtc
ccag*GTGGTCACAACCAGCCTTCCCTGTCAGCCTCGCCGAGCCCTGTGGTCCCCCTAGGAGGACATGTGACAC*
*TTCGCTGTTATTCCCACCTTTCATTTGTCATAG*TCAAACTATCCAAAAGAGTTGGGAGCAACATCTCTGAGAT
CTACAGTGTCCTTTCTCACAACACCTTTGCCTTCAACCCGGTCACCACAGCACATGCAGGGACCTACAGATGT
TCTGTATCTTACACCCAGAATCCCTCTGTGTGGTCAGCACTCAGCGAACCCCTGGAGATCATGGTCACAGgta
ggagaagcccatcccagcccatgtccacctgtgtccagatggcccttgtcagagtccacacccagcgtaccca
gagagtcattgtaggattcctggccaggcctaactgtggacaagaggccatctgacaatttagagagaggata
tttaatgtgaggagttgggtacccaggtgaagagggaatgaggcagtgaagaaagaggagaatctacctagtg
attagagagaatagagtgtgggtgccaggtacaagaagagaggacccaaggaggagtggtgtcccctgagccg
cctgaccccagcaccagccttcctacgagggaagttgttattttgtcatagaggagccagagccttggggaag
acacggcccccctgggagatgcctctagaggcttggttggggcatggatggggcaatgttaccttcctcccctc
ctcctgccaagtctcctgacagtcatctcctttgctctagtgaatgtgcagaatgtctccaggaaagttagga
accacatcctacagggtcaccctggcccaccctatggagctgggaggggatgggtcgtggagtgggcctcaca
gtctgagagcctagaaagaacaaagaaggtgttgtggccattgtccaggtgagagtaaagcaggttgaggtga

ggcagggagatccaggatacaaaagacaccatccagatgatgaaggatgatgcccagagaaaatgcaagctca
cagtcaggagagacaggcaggctttggtgatgacggagaaacttcactcctgtgttctcagggcacgggctga
ggcaggaggacacctataatctcacggtctctttgctccagGTGTGTTCAGGAAACCCTCCATCTCAGCATTC
CCAAGCTCCTTAGTGCACACAGGAAGCAGTGTGACCCTGAGCTGTCTCTCAGAACTGGACTTTGATAAATTTA
TCCTGCACAAGGTTGGACCACACAGCATTCCCAGCTGCCTGCCAAGAGGGTCCATGCTGAGCTCCCCTACAC
CCAGGCTGACTTCTCCATAGATACCATGATGCCCACCCATGCAGGGACCTACAGATGCTACGGTTCTCTCAGT
CACTCTCCCTGTCAGTGGTCAGCTCCCAGTGACCCCCTGGACGTTGTGATCACCGgtgagtgtggccagacca
gtccctggtcttttcatgctctataggcttcaggggggtgacatggttattgatcacaatgttagtgcagtcag
aaaaaaacaggcacaagacataccgtgaacctctgtagaaaggggtgggggtgcaacaaggaaaagtccaacc
aacatagaatgtattataactaaagagaaaacaaattagtcagtgacagacacggtgaatgacatactaagag
agatacatgaacctagagtcgggactggattcccaccactcaggccctgaggggccttttggggtcaggttct
caccctgaaaccgcaggcccgttcctctccaccaggagacacagtgacacacacgtcccccagccagggccct
gggggatcttaatggagatggggaccctgaggagttgctcagcagtgtggtgtgactcgtccttctgcccaaa
gagggagaagtggtcacttgcaacctccccaagaacaggcacacctaacaccttgggaggtgtggatacacca
gccggttccagtggactcagcctgtgatgttctgacagatcctgtggagattattgcacttttcagcatgtaa
agttggaaacagtgagacccatagcctcatagctccaggtcagggaacgtaaggccagtgccatgttttctga
ggctctcaaaagaagataattatttccatgttccaggagggcctcacactatatatgggtttaaggggcagta
agacatgaagacacagtaaaaaaataatggaaaattaatgaaaaccagaagaattacaaagacccacaacgtg
agtcaagattgcagagactaaaatgttgttgagagaagcactagcagagctagagtttggtcccaagacaaga
ccaggcagagagaatttttttgttctttcactcaggataatgtgatatgttttgtgttgtaaaatcaaactctc
tttttctcctagGACAATATGAAAAGCCTTCTCTCTCCACCCATGTGGGCCCCACGATGGGCTCAGGAGAGAA
CATGACCTTGTCCTGCAGCTCTAAGACCCGATTTGACAAGTACCATCTGTCCAGGGAGGGGGAGGCCCATGAC
CGCTGGCTCAGTGAACGCCAGAGCCACAATGGAACATTCCAAGTCTACCTACCTCTGGGCTCTGAAACCCCTT
CCCACGGAGGGACCTATAGATGCTACGGCTACTTCTATTTCTCCCCCCATAAGTGGTCATCCCCAAGTGACCC
CCTGCACCTTTCAGTCACAGgtgaggatcccatacctgctgcataccctgggacctactgcatcatagagctg
tgtctgaggggtgttctgatgagacacagggatcttagaatctggacaaatgacccatgagaaatgcagagag
cgaaacagaagctgtggaggagagggactgtcagtttaggaaggtaaagaagtcacctccaaatctccatctt
cctcctgtacagaggccacttggggggtcctgcatacccaggtgagtgaggaaagggctcaggaggaggtgagg
ccagatgcagtttgggaagatcagaggttgcatccaccgctcagctttatccgtcttccagaagccattcctc
acctctcccccacagacagagctccctgctgagaaacgtgggtattatcaccccaaaaggagaaaacatctct
tgattatgggctccttcttcatcaggcatctcctagtgtttgctgcacattcacgtcccagagggagggagtg
tccctgcccctgggatcctgtgagatggtctgcagggcccaaaccagtcagtaggtgaagacagagtgcaggg
aggacaggggaccgacattgccctagactgtagacagtgatcctagctcactggtgcaaggaatgacccttgc
tgacccccttctctgtccttttccagGAACCCCTAAGAGTACTTGTCTATCACCCATAGAATCAACCTCCAAAT
CCAgtaagtaaaggaggctcttagcttcatgtgtgaatccctggggagacagaaggctcgtgtgtgcgtgatg
gctttgccacctcccagccctaggaccgtggtctggtcaactccaatgtctgcttcatagattctcgatgggc
atttgagacactgtggtcagcgtggggatcagtggtggcatttagactcacgccctgggatcagggtctcatc
caaaagggaggaaggggccagagggaacctgagcactccagagaagtgatgcttgtcatccctccatcaaggt
ctgtgaaaatgggagtgtggtccatatttaatgtcctatgatctgaatactcaagtttagatgcgaaagtgt
cctgtattcataagaatttcattccagtgtcatgtgagggaggagaaaaatcataacaataaattaattaat
tgatttaaaattccttaacttcatattcattaagatggtcatgtgataaaaaaagccttagtgtatcatttag
aacggttggtagggaaggtcctacagaggaggtgacatttaaggcattatctctgtgacaaggggagccagtg
ttgagaatactggtggaaggactttcccagcagttggaacagcgcgtgccctgaacttaacctggcagtggct
tggtgtgttcacagcacccatgtggctgcgtgtggtcggggaggtggagttgatagaactagacaggagtcag
cttgcttacgggaaggattctaagcaatcataaagagtttcaattgtattctggccaagatgtgaagccagta
agagatttcaatcagaaaaatgtcatgatatgcatcctatttcagaaataaaaattgccagatatgaaaagaa
attattttagggaaacaggagtggaatgtgtatgactggggaagtcttcaacatccttgtagagaaaattcat
ggtggcttagacatgaaagtgccagagaaggagttgagaaattgagttcaggacacactcagagctggactca
ttaagagggagatttcagaacctagatctggtatgtctcagcagagtggcgttggacatactcatttctctct
aagcctctgtttcagttgatggaaactatacttgacctatcagagaaagaaatagctcaacaaggattctagt
gagggaacagacatcatccttgaaagagaaagactgaagtcataatcttgggcataagtgatggctctgctgg
ttctagtcacttaatgtcacaaagcaacaatttccctaaacaggacactaggctaatggaccctccttccttc
tgccaagcataactgtagtcacaaatgtgaaagtcctcttgcctccagaaggggtcctagtacagagaatgtg
ctcatatatgacacatcccattcccatcccacttcttctctctttaatactccacccaccccactcttagtta
tgactgggagggagtattaatatgctgtgtcgcaagttcaaatctcagcaaccagcactagaaacatcctcag
ctccaagaaagtacggttgtgccagagtcaggaggaccaaggggaaatggtgatcagctatggtagatacaga
gagtttccttttcatgcaaaaacgaactgccttgtctcatgaggatgtgggggagtggggcacatttcagcct
ggtagatctagattcagacctctgcatatctcttcctggttgattgatttggaaaactgtcttcactgctctt
tgaatttttttcatctgttggaaactggagaaagccaatgttcagttttaggaatgttggggagtccaacat
agagtgcatgatgagtctagcatgtactggcacaaaataggcaaccagccaattggagcttttgttgttcgct
tgttgacagAAGCAGGTCCACCTCCAGGACAGTTCCATAAACAGGACATTCTCATTGGACTCTCAGGAGTCAT
CATCTCCATTGGCATTTTCCTCTCTGTTCTTCTTGGTTACTGGTGTTCTACCAAAAATCgtaagtctgaaacg
ggagaggacagtatcatgtgagtggagtggggtttgtttgggacacaaaggtgcatgttcacttcctgtgggt
tgatcgtgcttctgtcgaggttctttttggaggatgcctaccccttttccttaacggaccaaccagccttccct
tgcccttcctcctccccagctctggggaggccactggtgtgcaagaagggatggtccagaagagaggcagtag
gatggggcacaggactggaggggaccctggctcagtcacggtcaacccagagttgttgatagggcagttctct

gaatttagtgggagagactggctgagaaaccccgggcacaccctggtctcattctggtctcgtgtctctatgt
atgatatgaggatctggaagagcctctcaagagccttggctccctcttttatcccacagATCCTGCCATCATG
GACACAGAGCCCATGGAAGTCCAAATAATGGAGAGAAATgtgagttctcgggcccaacccctcactacagttt
catcctctccatatataaaataaccttccctttaattgttaaatatcaccctcttctcacctgtcgcctctca
gGACCCTGCAGCAGAAGAAACACAGGAGATAGTATATGCTCAGCTAAACCAGCACGCACTCACACGGAGAGAA
TTCAGTCTCACTTCCCAGTGTTCCAGATACCGCTTGGATGAGCCCAGTACCTGGGTGGACCTCCACACTTGCC
ACGTGGACCACGCTGAGGCCAGACCTGACCCTGGTCTCTGAacatgcagagcattgccacgcaaggtcctggt
tccagcccccggaggatttcctccatggacgctcatcctccttctcaaaccatgacaacatctctgggtgaca
acagtagagtcagaatacttgtggatcatcttaaaatcccagaggaccttcctagacacccagctattctagg
actctattttgagtaacctgtcatttaaggaggttactcacccacacattctgcacatctcattattaccgtc
tttttatgaaactctatgaagtgattctagaaagcttgatgtgtatgctcagccagtttagtcttcaaattac
tcaataaagttttgaagagatgaaatgtattcgacatcactacttttgaatgtagtagccacgagccacctgt
ggctattttaattaaaattggaatagattaaattaaataaa
