# Supplementary material for: A Novel System of Polymorphic and Diverse NK Cell Receptors in Primates
Source: PLoS Genet. 2009 Oct 16;5(10):e1000688. doi: 10.1371/journal.pgen.1000688 (PMC2757895; doi:10.1371/journal.pgen.1000688)
Supplement: Figure S2 — Multiple sequence alignments of CD94 proteins. (A) Multiple sequence alignment of mouse lemur, ruffed lemur, human, and common marmoset CD94 proteins. Sequences are subdivided into cytoplasmic (CY), transmembrane (TM), stalk, and C-type lectin-like domain (CTLD). (B) Amino acid sequence identities (in %) of mouse lemur, ruffed lemur, human and common marmoset CD94. Mimu, Microcebus murinus; Vava, Varecia variegata; Hosa, Homo sapiens; Caja, Callithrix jacchus. (0.01 MB PDF) [file pgen.1000688.s002.pdf]

A

```
                CY              TM
Mimu-CD94-1     MAVSQTTRWR      LISGTLGVICLLLMVTLGMLL 31
Mimu-CD94-2*01  ..........      ..................I.. 31
Mimu-CD94-2*02  ..........      ..................... 31
Mimu-CD94-3*01  ..........      ..................... 31
Mimu-CD94-3*02  ..........      ..................... 31
Vava-CD94-1     ..........      ........V....LA...I.W 31
Vava-CD94-2*01  ..........      ..............A...I.W 31
Vava-CD94-2*02  ..........      ..............A...I.W 31
Vava-CD94-3*01  .......W..      ..............A...I.. 31
Vava-CD94-3*02  .......W..      .................AI.. 31
Hosa-CD94       ...FK..L..      .......I...S..A...I.. 31
Caja-CD94*01    ...FKA.F..      .......I...S..A...I.. 31

                stalk
Mimu-CD94-1     KSL--------PNEIDIKPTSSSLPNKELQEGSDC 58
Mimu-CD94-2*01  ...--------ILQTSTEA.H.PRL.--....... 56
Mimu-CD94-2*02  ...--------ILQTSTEA.H.PRL.--....... 56
Mimu-CD94-3*01  ..SNFESFLMAHTKT.VES...PG.DR.....P.. 66
Mimu-CD94-3*02  ..SNFESFLMAHTKT.VESA..PG.DR.....P.. 66
Vava-CD94-1     .LY-------------MELI..PGL.T........ 53
Vava-CD94-2*01  PIY-------------TE.I..PG..M........ 53
Vava-CD94-2*02  PIY-------------TE.I..PG..M........ 53
Vava-CD94-3*01  QP.-----FLPFTGLR.Q..L..GL.I..H..... 61
Vava-CD94-3*02  QP.-----FLPFTGLR.Q..L..GL.I..H..... 61
Hosa-CD94       .NS--------FTKLS.E.AFTPG..I...KD... 58
Caja-CD94*01    .NS--------FTKLS.ESAFTPG..I...KD... 58

                CTLD
Mimu-CD94-1     CSCPEKWVGYRCNCYFISLEMKTWNESRNFCVSQNSSLLQLQNRDELPFMHSNTKFYW 116
Mimu-CD94-2*01  ..................G.V...E...I..I...............A..Y..QY... 114
Mimu-CD94-2*02  .....E............G.V...E...I..I...............A..Y..QY... 114
Mimu-CD94-3*01  ....K......W......S............A...............N..NFSQR... 124
Mimu-CD94-3*02  ....K......W......S............A...............N..NFSQR... 124
Vava-CD94-1     ..................S.V...E.....LF..............A.TQFSQQ... 111
Vava-CD94-2*01  ..................S.....E......................R..QLSRR... 111
Vava-CD94-2*02  ..................S.....E......................G..QLSPR... 111
Vava-CD94-3*01  ..................S.V...E.....LFR.............N....GQH... 119
Vava-CD94-3*02  ..................S.V...E.....LFR.............N....GQH... 119
Hosa-CD94       ...Q..............S.Q.......HL.A..K........T...D..S.SQQ... 116
Caja-CD94*01    ...Q...I..........S.SR......HL.A..K.......S....D..S.SQQ... 116

                CTLD
Mimu-CD94-1     IGLSYNTERGVWQWENGSALSQNLFSSFETPDPENCILYDLTNNTMDEPCERKNHYIC 174
Mimu-CD94-2*01  ..........D....D..NF.R...........K...V..AMKSAV......Q..F.. 172
Mimu-CD94-2*02  ..........D....D..NF.R...........K...V..AMKSAV......Q..F.. 172
Mimu-CD94-3*01  ......E...A.L......F.WD....L....TK...V.KPM.SA.......E..... 182
Mimu-CD94-3*02  ......E...A.L......F.WD....L....TK...V.KPM.SA.......E..... 182
Vava-CD94-1     ......E...D.L..D...P.WD....L.....N..VA.EPIISAV......E..... 169
Vava-CD94-2*01  ......E...D.L..D..TP.RD....LV.S..K...A.KPM.GVV......E.Y... 169
Vava-CD94-2*02  ......E...D.L..D..TP.RD....LV.S..K...A.KPM.GAV......E.Y... 169
Vava-CD94-3*01  ......E...D.L..D..T........L.....K...V.KPMKSAV......E..... 177
Vava-CD94-3*02  ......E...D.L..D..T........L.....K...V.KPMKSAV......E..... 177
Hosa-CD94       .....SE.HTA.L.........Y..P....FNTK...A.NPNG.AL..S..D..R... 174
Caja-CD94*01    .....SE.QNA.L..S..V...D..PL.G.SNRK...A.NPKG.AV.....I..RF.. 174

                CTLD
Mimu-CD94-1     ILQLI*                            179
Mimu-CD94-2*01  KKS.FKCFLGQRGGDNQSSITTNSIFTPHYCY* 204
Mimu-CD94-2*02  KKS.FKCFLGQRGGDNQSSITTNSIFTPHYCY* 204
Mimu-CD94-3*01  KQ...*                            187
Mimu-CD94-3*02  KQ...*                            187
Vava-CD94-1     KQ.H.*                            174
Vava-CD94-2*01  KK.H.*                            174
Vava-CD94-2*02  KK.H.*                            174
Vava-CD94-3*01  KK...*                            182
Vava-CD94-3*02  KK...*                            182
Hosa-CD94       KQ...*                            179
Caja-CD94*01    KQ.V.*                            179
```

B

	Mimu-CD94-2*01	Mimu-CD94-3*01	Vava-CD94-1	Vava-CD94-2*01	Vava-CD94-3*01	Hosa-CD94	Caja-CD94*01
Mimu-CD94-1	76,8	77,1	71,8	74,7	75,4	66,5	61,5
Mimu-CD94-2*01		75,7	76,7	76,7	79,1	63,8	62,1
Mimu-CD94-3*01			79,3	79,9	75,8	70,4	66,5
Vava-CD94-1				83,3	80,5	67,2	64,9
Vava-CD94-2*01					80,5	67,8	66,7
Vava-CD94-3*01						68,7	65,4
Hosa-CD94							87,2
