# Supplementary material for: A Novel System of Polymorphic and Diverse NK Cell Receptors in Primates
Source: PLoS Genet. 2009 Oct 16;5(10):e1000688. doi: 10.1371/journal.pgen.1000688 (PMC2757895; doi:10.1371/journal.pgen.1000688)
Supplement: Figure S3 — Multiple sequence alignments of NKG2 proteins. (A) mouse lemur and (B) ruffed lemur NKG2 and Ly49L amino acid sequences. Sequences are subdivided into cytoplasmic (CY), transmembrane (TM), stalk, and C-type lectin-like domain (CTLD). ITIM are highlighted in green, YxxM motif in yellow and positively charged amino acids in the TM in red. An ITIM at unusual position was found in mouse lemur NKG2-5 and is marked in blue. It is not clear whether this motif functions as an ITIM. Due to failure of obtaining a 5′-RACE product for ruffed lemur Ly49L, the corresponding deduced amino acid sequence is incomplete at the amino terminal end. The two identified ruffed lemur Ly49L alleles show only a single synonymous substitution and have therefore identical deduced amino acid sequences. Mimu, Microcebus murinus; Vava, Varecia variegata. (0.02 MB PDF) [file pgen.1000688.s003.pdf]

A

```
                 CY
Mimu-NKG2-1*01   MDNQRVIYSEMNLAKNPKRQQRKSKDTKSSISKTEQEITYAELNLQNAAQ-DLQGDDR 57
Mimu-NKG2-1*02   ..................................................-....... 57
Mimu-NKG2-2*01   .K..K.T..G.....D.......F.GD.....V......QV.......TSG....KEK 58
Mimu-NKG2-2*02   .K..K.T..GL....D.........GD.....V......QV....H..TSG....KEK 58
Mimu-NKG2-2*03   .K..K.T..GL....D.........GD.....V......QV....H..TSG....KEK 58
Mimu-NKG2-2*04   .K..K.T..GL....D.........GD.....V......QV....H..TSG....KEK 58
Mimu-NKG2-3*01   ....T.....IL...T.....K.....T....E.D.............-...RV.. 57
Mimu-NKG2-3*02   ....T......L...T.....K.....T....E.D.............-...RV.. 57
Mimu-NKG2-3*03   ....T......L...T.....K.....T....E.D.............-...RV.. 57
Mimu-NKG2-5*01   .K....-----....D.........GG.....V.....AQV.......TL-..S.KEK 52
Mimu-NKG2-5*02   .K....-----....D.........GG.....V.....AQV.......TL-..S.KEK 52
Mimu-NKG2-5*03   .K....-----....D.........GG.....V.....AQV.......TL-..S.KEK 52
Mimu-NKG2-8*01   ..............Q.S..............E.K...............-....... 57
Mimu-NKG2-8*02   ..............Q.S..............E.K...............-....... 57
Mimu-NKG2-8*03   ..............Q.S..............E.................-....... 57

Mimu-Ly49L*01                               MSDQGVIYSTVRFLQSSSESESGRSPDVTLRP 32
Mimu-Ly49L*02                               ......................Q......K. 32

Mimu-NKG2D*01                                      MNEFHNYNWKLAKRDTSTQWKKQRS 25
Mimu-NKG2D*02                                      ......................... 25


                 CY                            TM
Mimu-NKG2-1*01   SYHCKDLLLPPEK                 LIAGILGIICLILMPM-VIK---LVI 92
Mimu-NKG2-1*02   .............                 ..............S.-...---... 92
Mimu-NKG2-2*01   TCYYQFS......                 .T.E..AV..TV.VSS-.L.MI-.LA 95
Mimu-NKG2-2*02   TCYYQFS......                 .T.E..A...TV.VGS-.L.MI-.IA 95
Mimu-NKG2-2*03   TCYYQFS......                 .T.E..A...TV.VGS-.L.MI-.LA 95
Mimu-NKG2-2*04   TCYYQFS......                 .T.E..A...TV.VSS-.L.MI-.LA 95
Mimu-NKG2-3*01   ...Y......Q..                 ........L..V.IMST.V-TS-..- 93
Mimu-NKG2-3*02   ...Y......Q..                 ........L..V.IMST.V-TS-..- 93
Mimu-NKG2-3*03   ...Y......Q..                 ........L..V.IMST.V-TS-..- 93
Mimu-NKG2-5*01   ICYY.LS......                 .T.E..AV..VV.VGS-.L.M-V.IA 89
Mimu-NKG2-5*02   ICYYNVS......                 .T.E..AV..VV.VGS-.L.M-V.IA 89
Mimu-NKG2-5*03   ICYY.LS......                 .T.E..AV..VV.VGS-.L.M-V.IA 89
Mimu-NKG2-8*01   .............                 ...........V.IMST.V-TS-..- 93
Mimu-NKG2-8*02   .............                 ...........V.IMST.V-TS-..- 93
Mimu-NKG2-8*03   .............                 ...........V.IMST.V-TS-..- 93

Mimu-Ly49L*01    EKTDDKEFSVSWR                 LIAVSLGILCLVLLVIVTVLVT 67
Mimu-Ly49L*02    G............                 ...................... 67

Mimu-NKG2D*01    TLITSRCTENSSP                 FFLCRSIAIAMGIHFIIMVTIL 60
Mimu-NKG2D*02    K............                 ...................... 60

                 stalk
Mimu-NKG2-1*01   --PS--RLMQNNSLQNMRTQKDSDSSAYHCAH 120
Mimu-NKG2-1*02   --..--....K..................... 120
Mimu-NKG2-2*01   HI.CTLTQKP.I.F.TI...-------..G.GR 122
Mimu-NKG2-2*02   HI.CTLTQKP.I.F.AI...-------..G.GR 122
Mimu-NKG2-2*03   HI.CTLTQKP.I.F.TI...-------..G.GR 122
Mimu-NKG2-2*04   HI.CTLTHKP.I.F.TI...-------..G.GR 122
Mimu-NKG2-3*01   -NS.RVPQE...FS.TT...------.CN.GR 119
Mimu-NKG2-3*02   -NS.RVPQE...FS.TT...------.CN.GR 119
Mimu-NKG2-3*03   -NS.RVPQE...FS.TT...------.CN.GR 119
Mimu-NKG2-5*01   LI.FTVIKK.D..F..VK...-------..D.GR 116
Mimu-NKG2-5*02   LI.FTVIKK.D..F..VK...-------..D.GR 116
Mimu-NKG2-5*03   LI.FTVIKK.D..F..VK...-------..D.GR 116
Mimu-NKG2-8*01   -Y..TETKK...T..TV...---------...GR 119
Mimu-NKG2-8*02   -Y..TETKK...T..TV..P.--------...GR 119
Mimu-NKG2-8*03   -Y..TETKK...T..TV...---------...GR 119

Mimu-Ly49L*01    KIFQCIQERHQQQEILGHLSQKDNYLKEQLLINKTLEYDILKNESLQQKKKLDSLFLKNN 127
Mimu-Ly49L*02    .........H.................................................. 127

Mimu-NKG2D*01    SAIFINSLFNQGVPISLEGSYCGP 84
Mimu-NKG2D*02    ........................ 84
```

```
                 stalk                        CTLD
Mimu-NKG2-1*01                                CPEEWFTYSTNCYYIGKELKTWDESVTACAS 151
Mimu-NKG2-1*02                                ..........................M.... 151
Mimu-NKG2-2*01                                .....LA........S..T....D....... 153
Mimu-NKG2-2*02                                .....LA........S..T....D..K.... 153
Mimu-NKG2-2*03                                .....LA........S..T....D..K.... 153
Mimu-NKG2-2*04                                .....LA........S..T....D....... 153
Mimu-NKG2-3*01                                .....L.........S..T....D..K.... 150
Mimu-NKG2-3*02                                .....L.........S..T....D..K.... 150
Mimu-NKG2-3*03                                .....L.........S..T....D..K.... 150
Mimu-NKG2-5*01                                .L.............S..F......LM.... 147
Mimu-NKG2-5*02                                ...............S..F......LI.... 147
Mimu-NKG2-5*03                                ...............S..F......LI.... 147
Mimu-NKG2-8*01                                .............S.S..F............ 150
Mimu-NKG2-8*02                                .........S...S.S..F............ 150
Mimu-NKG2-8*03                                .........S...S.S..F............ 150

Mimu-Ly49L*01    ICHIKNEIFSKSLENT             GKRYEAHWTCCGLSCYYFAMENKNWKGCKQT 174
Mimu-Ly49L*02    ................             ...................V........... 174

Mimu-NKG2D*01                                 CPKNWLCYRNNCYQFFNESKNWYESQASCMS 115
Mimu-NKG2D*02                                 ............................... 115


                 CTLD
Mimu-NKG2-1*01   NNSNLFYTENEEEMKFLGSLSLLSWFGVSRNSSDHPWVLRDGSTFKLNIIETEYGNRNCA 211
Mimu-NKG2-1*02   ......................................................D..... 211
Mimu-NKG2-2*01   K....L.ID........S...KKA.I..F....EQ...S.N......K.E.SIP.RH... 213
Mimu-NKG2-2*02   K....L.ID........S...KKA.I..F....EQ...S.N......K.E.SIP.KH... 213
Mimu-NKG2-2*03   K....L.ID........S...KKA.I..F....EQ...S.N......K.E.SIP.EH... 213
Mimu-NKG2-2*04   K....L.ID........S...KKA.I..F....EQ...S.N......K.E.SIP.KH... 213
Mimu-NKG2-3*01   K....L.ID........S...KKA.I..F....EQ..IS.N......K.E..VS.RH... 210
Mimu-NKG2-3*02   K....L.ID........S...KKA.I..F....EQ..IS.N......K.E..VS.RH... 210
Mimu-NKG2-3*03   K....L.ID........S...KKA.I..F....EQ...S.N......K.E..VS.RH... 210
Mimu-NKG2-5*01   KS.S.L.IDD...........NQA.I..F..G..R...SIN....N.K.E.IL..RNH.. 207
Mimu-NKG2-5*02   KS.S.L.IDD...........NQA.I..F..G..R...SIN....N.K.E.IL..RNH.. 207
Mimu-NKG2-5*03   KS.S.L.IDD...........NQA.I..F..G..R...SIN....N.K.E.IL..RNH.. 207
Mimu-NKG2-8*01   .....L.ID.A..II.....LRQ..I..F....H....SIN......Q.V..AT.K.... 210
Mimu-NKG2-8*02   .....L.ID.A..II.....LRQ..I..F....H....SIN......Q.V..AT.K.... 210
Mimu-NKG2-8*03   .....L.ID.A..II.....LRQ..I..F....H....SIN......Q.V..AT.K.... 210

Mimu-Ly49L*01    CQSYKSSLLKIDDEDELTFVQLQIYKNNYWIGLSYDERESKWKWVDSGSSPGINVGIMNS 234
Mimu-Ly49L*02    .R.......................................................... 234

Mimu-NKG2D*01    QNSSLLKIYSRVDQDFLKLVKSYHWMGLVQMSTNGSWQWDDGTILSPNQLTVIEMQRGSC 175
Mimu-NKG2D*02    ............................................................ 175


                 CTLD
Mimu-NKG2-1*01   VLYKYRLQSDECRSSKLYICKHKR* 235
Mimu-NKG2-1*02   L.......................* 235
Mimu-NKG2-2*01   ..QSPS.H.GG.E.T.T.....EF* 237
Mimu-NKG2-2*02   ..QSPS.H.GG.E.T.T.....EF* 237
Mimu-NKG2-2*03   ..QSPS.H.GG.E.T.T.....EF* 237
Mimu-NKG2-2*04   ..QSPS.H.GG.E.T.T.....EF* 237
Mimu-NKG2-3*01   ..QSPS.H.GG.E.T.T.....EP* 234
Mimu-NKG2-3*02   ..QSPS.H.GG.E.T.T.....EP* 234
Mimu-NKG2-3*03   ..QSPS.H.GG.E.T.T.....EP* 234
Mimu-NKG2-5*01   ...SSS.H.SG...P.T......L* 231
Mimu-NKG2-5*02   ...SSS.H.SG...P.T......L* 231
Mimu-NKG2-5*03   ...SSS.H.SG...P.T......L* 231
Mimu-NKG2-8*01   I.HSDG....G.E.L.R.H....L* 234
Mimu-NKG2-8*02   I.HSDG....G.E.L.R.H....L* 234
Mimu-NKG2-8*03   I.HSDG....G.E.L.R.H....L* 234


Mimu-Ly49L*01    SSGRGKCGFLSSTRVAAIDCIQTYNCICEKRIGCSIFSASACTEKKR* 281
Mimu-Ly49L*02    .......A.......................................* 281

Mimu-NKG2D*01    VVYGSSFKGYTENCSTPYMYICMRRIM* 202
Mimu-NKG2D*02    .....N.....................* 202
```

B

```
                CY
Vava-NKG2-1     MDNQRVIYSEMKLAKNPKRQQRKSKDTKSSISETEWEITYAELNLQNAAQDLRGDDTSYH 60
Vava-NKG2-2     .K....T..G.N...D.........GK.....V..Q...QV.......PL..Q.K.KTCY 60
Vava-NKG2-3     .N........LN......Q...ET.G.........R....V..........PQ...K... 60
Vava-NKG2-4     .K....T..G.N...D.........GG...T.V..Q..IQV...F...PL..Q.KEKTCY 60
Vava-NKG2-5     .K....T..G.N...D.........GG.....V..E...QV...F...TL..QEKEKTCY 60
Vava-NKG2-6*01  .K....T..GLN...D..M......GG...F.V..Q...PV....E..TL..Q.N.N.CY 60
Vava-NKG2-6*02  .K....T..GLN...D..M......GG...F.V..Q...PV....E..TL..Q.N.N.CY 60
Vava-NKG2-7     .K..T.T..G.N...D.....T...GG...T.V..Q...QV.......PL..Q.KEKTCY 60
Vava-NKG2-8     ...E.......N....S.S......G.........QK...............Q...N... 60

Vava-Ly49L                                     IYSTVRFVRSPSESGSEQRSDVTQRPGKT 29

Vava-NKG2D                                      MNKVHNYNLKLAKHNTCTQWQKQRSPLI 25


                CY                        TM
Vava-NKG2-1     CKDLLLPPEK                LIAGILGIICLVLMFM-VA--GVLIA 93
Vava-NKG2-2     Y.FS....G.                .T.E..AV..I..VGS-.LKMI-... 94
Vava-NKG2-3     ..........                ............FTSA-.V-TR--LV 92
Vava-NKG2-4     Y.FS......                .T.E..AV..I..VGS-.LKM-.... 94
Vava-NKG2-5     Y.FS..S.G.                .T.E..AV..I..VGS-.LKMI-... 94
Vava-NKG2-6*01  R.FS......                .T.E..AV..I..VGS-.LKM-...V 94
Vava-NKG2-6*02  R.FS......                .T.E..A...I..VGS-.LKM-...V 94
Vava-NKG2-7     Y.FS....G.                .T.E..AV..I..VGS-.LKMI-... 94
Vava-NKG2-8     ..........                ..............SV-IIKC---VI 92

Vava-Ly49L      GDKVFSVSWR                FIAVSLGILCLLLLMIVIVLVT 61

Vava-NKG2D      TSKCTENSSP                YFLCRSIAIALGIHFIIMVTIL 60


                stalk
Vava-NKG2-1     ---SMVKEMQNNSLQTMGTQKAYNCSH 117
Vava-NKG2-2     RIPFTLTQKP...S...R.....D.GR 121
Vava-NKG2-3     -NP--LPQN....SK.IR....C.RG. 116
Vava-NKG2-4     LIP--LTQ--.S.S..V......Y.GR 117
Vava-NKG2-5     RIP----LKL.S.S..IR.....D.GR 117
Vava-NKG2-6*01  LIPFTIM.K....F.NVK.....D.G. 121
Vava-NKG2-6*02  LIPFTIM.K....F.NVKI....D.G. 121
Vava-NKG2-7     RIPFTLTQKP...F..IR.H.....G. 121
Vava-NKG2-8     --P--LPQK..D.S..TR......... 115

Vava-Ly49L      KMFQCIQERHQQEILRDLSQNDNYLKEQLLTNKTLEYDILKNESLQQKKELDSLFLKKN 120

Vava-NKG2D      SAIFINSLFNQGVPISLKESYCGP 84


                stalk                        CTLD
Vava-NKG2-1                                  CPEEWFTYSTNCYYIGKELKTWDESVTACAS 148
Vava-NKG2-2                                  ...............S..S............ 152
Vava-NKG2-3                                  ...............S..F.......M.... 147
Vava-NKG2-4                                  ...............S............... 148
Vava-NKG2-5                                  ..................F............ 148
Vava-NKG2-6*01                               ...G.........S........Y........ 152
Vava-NKG2-6*02                               ...G.........S........Y........ 152
Vava-NKG2-7                                  ...............S............... 152
Vava-NKG2-8                                  ...............S............... 146

Vava-Ly49L      ICHTKNDIFSKSLENT             GKRYEGHWSCCGLSCYYFTMENKNWKGCKQT 167

Vava-NKG2D                                   CPKNWVCYRNNCYQFFNESKNWYESQASCVS 115
```

```
                 CTLD
Vava-NKG2-1      QNSSLLYIDNEEEMKFLDSLSLLSWIGVSRKSHRHPWVSLRGSTFKLKIAETEYAERNCA 208
Vava-NKG2-2      ...............L.G.F.KQA....F.T.SH.....RN........K..I.GTHH.. 212
Vava-NKG2-3      ...N.....K.......G...GKT....F.NTSHY....RN....Q..VE.II.GKHH.. 207
Vava-NKG2-4      N.TN..........R..G...EK...E.FHN.SH.....RN.L......K..I.GKHH.. 208
Vava-NKG2-5      N.............R..G...KQA....F.N.SH.....RN........E..I.GKHH.. 208
Vava-NKG2-6*01   ...N..........I..G..LRQ.....F.N.SD......HS.A...Q....AH.K.... 212
Vava-NKG2-6*02   ...N..........I..G..LRQ.....F.N.SD......HS.A...Q....AH.K.... 212
Vava-NKG2-7      ......S........L.G..............R.......................H... 212
Vava-NKG2-8      N..N...........L.G...KQA....F.N.SH.....IN........K..I.GKHH.. 206

Vava-Ly49L       CRSYRSSLLKIDDEDELAFVQLQTYKNYYWIGLSYDEKERKWKWVDSGSSPGLNFAIMNL 227

Vava-NKG2D       QNSSLLKIYSRVDQDFLKLVKSYHWMGLVQMSTNGSWQWEDGTILSPNQLTVIEMQKGTC 175


                 CTLD
Vava-NKG2-1      MLYKRRLQRDECGSSKPYICKHKF* 232
Vava-NKG2-2      A.DSL...STG...N.T.....EL* 236
Vava-NKG2-3      V.QSL...SAG.....R......I* 231
Vava-NKG2-4      A.DSL...SS....N.T.....EL* 232
Vava-NKG2-5      A.DSLT..SAG.....T......I* 232
Vava-NKG2-6*01   I.HSD...SNG....NR.H....L* 236
Vava-NKG2-6*02   I.HSD...SNG....NR.H....L* 236
Vava-NKG2-7      ........S...............* 236
Vava-NKG2-8      A.DSLN..SAG.....A.....EL* 230

Vava-Ly49L       TSGRGQCAFLSSTRVAVIDCIKTYNCICEKRIDCIFSASASTKKKR* 273

Vava-NKG2D       VVYGSSFKGYTENCSTPYMYICMRRIV* 202
```
