# Supplementary material for: A Novel System of Polymorphic and Diverse NK Cell Receptors in Primates
Source: PLoS Genet. 2009 Oct 16;5(10):e1000688. doi: 10.1371/journal.pgen.1000688 (PMC2757895; doi:10.1371/journal.pgen.1000688)
Supplement: Figure S4 — CD94 exon 4 to exon 5 sequence alignment of (A) potto (Perodicticus potto) and (B) Philippine tarsier (Tarsius syrichta). The shading threshold is 55% sequence identity. N in the sequences of potto CD94-6 and -7 and of tarsier CD94-9 denotes an unknown number of thymidine nucleotides. The CD94-7 sequence of the tarsier contains a stop codon in exon 4 which is highlighted in bold and underlined. Pepo, Perodicticus potto; Tasy, Tarsius syrichta. Alu elements are underlined. (0.03 MB PDF) [file pgen.1000688.s004.pdf]

A

```
             exon 4
Pepo-CD94-1  CAGAGAAGTGGGTTGGGTACCGATGCAACTGTTACTTCATTTCCACTGAAGAGAAAACTTGGGAGGGAAG 70
Pepo-CD94-2  CAGAGAAGTGGGTTGGGTACCGATGCAACTGTTACTTCATTTCCACTGAAGAGAAAACTTGGGAGGGAAG 70
Pepo-CD94-3  CAGAGAAGTGGGTTGGGTACCGATGCAACTGTTACTTCATTTCCACTGAAGTGACAACTTGGGAAGAAAG 70
Pepo-CD94-4  CAGAGAAGTGGGTTGGGTACCGATGCAACTGTTACTTCATTTCCACTGAAGTGAAAACTTGGGAAGAAAG 70
Pepo-CD94-5  CAGAGAAGTGGGTTGGGTACCGATGCAACTGTTACTTCATTTCCACTGAAGTGAAAACTTGGGAAGAAAG 70
Pepo-CD94-6                      CGAAGCAACTGTTACTTCATTTCTACTGAAGTGACAACTTGGGAAGAAAG 50
Pepo-CD94-7                      CGAAGCAACTGTTACTTCATTTCTACTGAAGTGACAACTTGGGAAGAAAG 50

                                                                 intron 4
Pepo-CD94-1  TAGGAAAGTCTGCATCTCTCAGAATTCCAGTTTGCTTCAGCTTCGAAACAAAGATGAACTGGCATGTACT 140
Pepo-CD94-2  TAGGAAAGTCTGCATCTCTCAGAATTCCAGTTTGCTTCAGCTTCGAAACAAAGATGAACTGGCATGTACT 140
Pepo-CD94-3  TAGGAAGTTCTGTGTTTCTCAGAATTCCAGTTTGCTTCAGCTTCGAAACAAAGATGAACTGGCACGTACT 140
Pepo-CD94-4  TAGGAAGTTCTGTGTTTCTCAGAATTCCAGTTTGCTTCAGCTTCAAAACGAAGAAGAACTGGCATGTACT 140
Pepo-CD94-5  TAGGAAGTTCTGTGTTTCTCAGAATTCCAGTTTGCTTCAGCTTCAAAACGAAGAAGAACTGGCATGTACT 140
Pepo-CD94-6  TAGGAAGTTCTGTGTTTCTCAGAATTCCAGTTTGCTTCAGCTTGGAAACAAAGATGAACTGGCATGTACT 120
Pepo-CD94-7  TAGGAAGTTCTGTGTTTCTCAGAATTCCAGTTTGCTTCAGCTTGGAAACAAAGATGAACTGGCATGTACT 120

Pepo-CD94-1  TGAATCTAGCCTCCTATATTTTCTTTGCTCT----ATCAAAGTATCTAAATAAGCTAAACACTTTGATTC 206
Pepo-CD94-2  TGAATCTAGCCTCCTATATTTTCTTTGCTCT----ATCAAAGTATCTAAATAAGCTAAACACTTTGATTC 206
Pepo-CD94-3  TGAATCTAGCCTCCTATATTTTCTTTGCTCT----TTCAAAGCATCTAAATAAGCTAAACACTTTGATTC 206
Pepo-CD94-4  TGAATCTAGCCTCCTATATTTTCTTTGCTCTGGATATCAAAGTATCTAAATAAGCTAAACACTTTGATTC 210
Pepo-CD94-5  TGAATCTAGCCTCCTATATTTTCTTTGCTCTGGATATCAAAGTATCTAAATAAGCTAAACACTTTGATTC 210
Pepo-CD94-6  TGAATCTAGCCTCCCATATTTTCTTTGCTCTGGATAGCAAAATATCTAAATAAGCTAAACACTTTGATTC 190
Pepo-CD94-7  TGAATCTAGCCTCCCATATTTTCTTTGCTCTGGATAGCAAAATATCTAAATAAGCTAAACACTTTGATTC 190

Pepo-CD94-1  AAGTCTTTAATCAGATTACAGGCAGTCTCCAGGTTATGAACGAGATAGGTTCTGTAGGTTTGTTCTTAGT 276
Pepo-CD94-2  AAGTCTTTAATCAGATTACAGGCAGTCTCCAGGTTATGAACGAGATAGGTTCTGTAGGTTTGTTCTTAGT 276
Pepo-CD94-3  AAGTCTTTAATCAGATTATAGGCAGTCTCCAGGTTATGAATGAGATAGGTTCTGTAGGTTTGTTCTTAGT 276
Pepo-CD94-4  AACTCTTTAATCAGATTACAGGCAGTCTCCTGGTTATGAACAAGATAGGTTCTATATGTTTATTGTTAGC 280
Pepo-CD94-5  AACTCTTTAATCAGATTACAGGCAGTCTCCTGGTTATGAACAAGATAGGTTCTATATGTTTATTGTTAGC 280
Pepo-CD94-6  AAGTCTTTAATTAGATTATAGGTGGTCTCCAGATTATGAACGAGATAGGTTCTGTATGTTTATTCCTAGT 260
Pepo-CD94-7  AAGTCTTTAATTAGATTATAAGTGGTCTCCAGATTATGAACGAGATAGGTTCTGTATGTTTATTCCTAGT 260

Pepo-CD94-1  TGAACCTGTTGCAAGTTGGAACAGGTACCTATTATCCCCATTCATAAGCGAGCCGCACGTCAGTGGGATG 346
Pepo-CD94-2  TGAACCTGTTGCAAGTTGGAACAGGTACCTATTATCCCCATTCATAAGCGAGCCGCACGTCAGTGGGATG 346
Pepo-CD94-3  TGAACCTGTTGCAAGTTGGAAGAGGTACCTATTATCCGCATTCGTAAGCGAGTCACATGACAGTGGGATG 346
Pepo-CD94-4  TGAATCCATTGCAATTTGGAACAGGTACCTATTATTCCCATTCGTAAGTGAGCCGCACGTCAGTGGGATG 350
Pepo-CD94-5  TGAATCCATTGCAATTTGGAACAGGTACCTATTATTCCCGTTCGTAAGTGAGCCGCACGTCAGTGAGATG 350
Pepo-CD94-6  TGAATACATTGCAAGTTGTAACAGGTACCTGTTATCCCCATTCGTAAGGGAGCAGCACGTCAGTGGGATG 330
Pepo-CD94-7  TGAATACATTGCAAGTTGTAACAGGTACCTGTTATCCCCATTCGTAAGGGAGCAGCACGTCAGTGGGATG 330

Pepo-CD94-1  TTCGTAGCTCAGGGACTCTCTGTAGTAAATAGGACTCATGACCTATCAATTCCATGTACCTTAAAACTAT 416
Pepo-CD94-2  TTCGTAGCTCAGGGACTCTCTGTAGTAAATAGGACTCATGACCTATCAATTCCATGTACCTTAAAACTAT 416
Pepo-CD94-3  TTTGTAGCTCAGGGACTCTCTGTAGTGAATAGAATTCATGACCTATCAATTTCATGTACCTTAAAACTAT 416
Pepo-CD94-4  TTTGTAGCTCAGGGACTCTCTGTAGTAAATAGAACCAATGACCTATCAATTCCATGTACCTTAAGATTAT 420
Pepo-CD94-5  TTTGTAGCTCAGGGACTCTCTGTAGTAAATAGAACCAATGACCTATCAATTCCATGTACCTTAAGATTAT 420
Pepo-CD94-6  TTTGCAGCTCAGGGACTCTCTGTAGTAAATAGAACTAATGACCTATCAATTCCATGTACCTTAAAACTAT 400
Pepo-CD94-7  TTTGCAGCTCAGGGACTCTCTGTAGTAAATAGAACTAATGACCTATCAATTCCATGTACCTTAAAACTAT 400

Pepo-CD94-1  CATTGTAAAACCCACATAAAATTCCTCAAAGAATTACATCATAGAGATAAAAATTAAGATAAGTGACA-- 484
Pepo-CD94-2  CATTGTAAAACCCACATAAAATTCCTCAAAGAATTACATCATAGAGATAAAAATTAAGATAAGTGACA-- 484
Pepo-CD94-3  CATTGTAAAACCCACATAAAATTCTTCAACGATTTACATCTTAGAGATAAAAATTAAGATAGGTGACAGA 486
Pepo-CD94-4  CATTGTAAAACCCACATACAATTCCTCAAAGAATTACATTTTAGAGATAAAAATTAAGATAGGTGACAGA 490
Pepo-CD94-5  CATTGTAAAACCCACATACAATTCCTCAAAGAATTACATTTTAGAGATAAAAATTAAGATAGGTGACAGA 490
Pepo-CD94-6  CATTGTAAAACCCACATAAAATTCCTCAAA-AATTACATCTTAGAGATAAAAATTAAGTTGGGTGACAGA 469
Pepo-CD94-7  CATTGTAAAACCCACATAAAATTCCTCAAAGAATTACATCTTAGAGATAAAAATTAAGTTGGGTGACAGA 470

Pepo-CD94-1  --AAGTGCGAGAAACTTTTCATGGAAAATTCAAAGAAGAAATAACAGGTCACTGCTTTGGACTTTGCAAT 552
Pepo-CD94-2  --AAGTGCGAGAAACTTTTCATGGAAAATTCAAAGAAGAAATAACAGGTCACTGCTTTGGACTTTGCAAT 552
Pepo-CD94-3  TAAAGTGCGAGAAACTTTTCAGGGAAAATTCAAAGAAGAAATAATAGGTCATTATTTTGGACTTTGCAAT 556
Pepo-CD94-4  G-AAGTACAAGCAATTTCTCAGGGAAAATTCAAAGAAGAAATAATAGTTCATTATTTTGGACTTTGCAAT 559
Pepo-CD94-5  G-AAGTACAAGCAATTTCTCAGGGAAAATTCAAAGAAGAAATAATAGTTCATTATTTTGGACTTTGCAAT 559
Pepo-CD94-6  TAAAGTGGGAGGAACTTTTCAGGGAAAATTCAAAGAAGAAATAACAGGTCATTATTTTGGACTTTGCAAT 539
Pepo-CD94-7  TAAAGTGGGAGGAACTTTTCAGGGAAAATTCAAAGAAGAAATAACAGGTCATTATTTTGGACTTTGCAAT 540

Pepo-CD94-1  GTTTAGTTCTTTCAATGGAATTTATAATTTCTTTTGTAACTATGTATCTTTTGGCATTTTTCTAGCTTAT 622
Pepo-CD94-2  GTTTAGTTCTTTCAATGGAATTTTTAATTTCTTTTGTAACTATGTATCTTTTGGCATTTTTCTAGCTTAT 622
Pepo-CD94-3  GTTTAGTTCTTTCAATAGAATTTATCATTTCTTTTGTAACTACATATCTTTTGGCATTTTTCTAGCTTAT 626
Pepo-CD94-4  TTTTACTTCCTTTAATGGAATTTATAATTTCTTTTGTAACTATACAGCTTTTGGCATCTTTCTAGGATAA 629
Pepo-CD94-5  TTTTACTTCCTTTAATGGAATTTATAATTTCTTTTGTAACTATACAGCTTTTGGCATCTTTCTAGGATAA 629
Pepo-CD94-6  TTTTAATTCTTTCAGTATAATTTATAATTTCTTTTGTAACTACCTATCCTTTGGCATTTTTCTAACTTAT 609
Pepo-CD94-7  TTTTAATTCTTTCAGTATAATTTATAATTTCTTTTGTAACTACCTATCCTTTGGCATTTTTCTAGCTTAT 610
```

```
Pepo-CD94-1   AACATGTAACAAGTATGGAAAGGATAATCATAAAAATTGCCCACTTCTT--------------------- 671
Pepo-CD94-2   AACATGTAACAAGTATGGAAAGGATAATCATAAAAATTGCCCACTTCTT--------------------- 671
Pepo-CD94-3   AACATGTAACAAGTATGGAAAGGATAATCATAAAAATTGCCCACTTCTT--------------------- 675
Pepo-CD94-4   AATATGAAAGAAGTATGGAAAGGATAATCATAGAAATTTCCCATTTCTTGGCTTGGCACCTGTAACTCAA 699
Pepo-CD94-5   AATATGAAAGAAGTATGGAAAGGATAATCATAGAAATTTCCCATTTCTTGGCTTGGCACCTGTAACTCAA 699
Pepo-CD94-6   AATATGTAACAAGTATGGAAAGGATAATCATAAAAATTACCCACATCTTNNNNNNNNNAAGAGACAAAGT 679
Pepo-CD94-7   AATATGTAACAAGTATGGAAAGGATAATCATAAAAATTACCCACATCTTNNNNNNNNNAAGAGACAAAGT 680

Pepo-CD94-1   ---------------------------------------------------------------------- 671
Pepo-CD94-2   ---------------------------------------------------------------------- 671
Pepo-CD94-3   ---------------------------------------------------------------------- 675
Pepo-CD94-4   GCAGCTAGAGCGCCAGCCACATACACCAGAGCTGGCAGGTTCAAATCCAGAACAACAATCACAACTAAAA 769
Pepo-CD94-5   GCAGCTAGAGCGCCAGCCACATACACCAGAGCTGGCAGGTTCAAATCCAGAACAACAATCACAACTAAAA 769
Pepo-CD94-6   TTCGCTTTATCGCCCTTGGTAGAGTGCCGTGTGGCGTCACACAGCTCACAGCAACCTCCAACTCCTGGGC 749
Pepo-CD94-7   TTCGCTTTATCGCCCTTGGTAGAGTGCCGTGTGGCGTCACACAGCTCACAGCAACCTCCAACTCCTGGGC 750

Pepo-CD94-1   ---------------------------------------------------------------------- 671
Pepo-CD94-2   ---------------------------------------------------------------------- 671
Pepo-CD94-3   ---------------------------------------------------------------------- 675
Pepo-CD94-4   CCAGAAAATAACCAGGCATTGTGGTGGGCACCTGTAGTCCCAACTATCTGGGAAACTGAGGTAAGAGAAT 839
Pepo-CD94-5   CCAGAAAATAACCAGGCATTGTGGTGGGCACCTGTAGTTCCAACTATCTGGGAAACTGAGGTAAGAGAAT 839
Pepo-CD94-6   TTAGGTTATTCTCTTACCTCAGCCTCCTAAGTACCTGGAACTACAGGCGCCTGCCACAACCGGCAGTTCG 819
Pepo-CD94-7   TTAGGTTATTCTCTTACCTCAGCCTCCTGAGTACCTGGAACTACAGGCGCCCGCCACAACCGGCAGTTCG 820

Pepo-CD94-1   ---------------------------------------------------------------------- 671
Pepo-CD94-2   ---------------------------------------------------------------------- 671
Pepo-CD94-3   ---------------------------------------------------------------------- 675
Pepo-CD94-4   CACTAAAGCCCAAGCATTTGAGTTTGCTGTGAGCTGTGATGCCACAGCACTCTGTCCAGGGTGACAACTT 909
Pepo-CD94-5   CACTAAAGCCCAAGCATTTGAGTTTGCTGTGAGCTGTGATGCCACAGCACTCTGTCCAGGGTGACAACTT 909
Pepo-CD94-6   GCCGGGACGGGGTTTGAACCTGCCACCCTCAGTATATGGGGCTGGTGCTCTACCCACTGAACCACAGGTG 889
Pepo-CD94-7   GCCGGGACGGGGTTTGAACCTGCCACCCTCAGTATATGGGGCTGGTGCTCTACCCACTGAACCACAGGTG 890

Pepo-CD94-1   ----------------------------------------AAGAAAAAGTGAA---TGTCTCCAGTTTCA 698
Pepo-CD94-2   ----------------------------------------AAGAAAAAGTGAA---TGTCCCCAGTTTCA 698
Pepo-CD94-3   ----------------------------------------AAGAAAAAGTGAAAAATGTCTCCCTTTTCA 705
Pepo-CD94-4   AAGACTCTATCTCAAAAAAAGAAACAATTGCCCACTTCTTAAGAAAAAGTGAATAATGTCTCCAGTTTCA 979
Pepo-CD94-5   AAGACTCTATCTCAAAAAAAGAAACAATTGCCCACTTCTTAAGAAAAAGTGAATAATGTCTCCAGTTTCA 979
Pepo-CD94-6   CTGCCCCA-----------------AATTACCCACTTCTTAAGAAACAGTGAAAAATGTCTCCAGTTTCA 942
Pepo-CD94-7   CTGCCCCA-----------------AATTACCCACTTCTTAAGAAACAGTGAAAAATGTCTCCAGTTTCA 943

Pepo-CD94-1   TGCAGTGTCTGTGCTGAGTTGAGGTCGAGTAGGTTATAACCCTGGGATTCATCCTTCATGATAGTGTTAA 768
Pepo-CD94-2   TGCAGTGTCTGTGCTGAGTTGAGGTCGAGTAGGTTATAACCCTGGGATTCATCCTTCATGATAGTGTTAA 768
Pepo-CD94-3   TACAGTGTTTGTACTGAGTTGAGGTTGAGTAGGTTATAATGCTGGGATTCATCCTTCATGATGGTATTAA 775
Pepo-CD94-4   TGCAGTGTCTGTGCTGAGTTGAGGT                                              1004
Pepo-CD94-5   TGCAGTGTCTGTGCTGAGTTGAGGT                                              1004
Pepo-CD94-6   TGCAGTGTCTGTGCTGAGTTGAGGT                                              967
Pepo-CD94-7   TGCAGTGTCTGTGCTGAGTTGAGGT                                              968

Pepo-CD94-1   TTTAGGATCAAATTTGATCATGGATCCCCTGTGAAGGAAGCTGTCATGAAAACTATCTTCCTTTAAGCTT 838
Pepo-CD94-2   TTTAGGATCAAATTTGATCATGGATCCCCTGTGAAGGAAGCTGTCATGAAAACTATCTTCCTTTAAGCTT 838
Pepo-CD94-3   TTTAGGATCAAATTTGATCATGGATCCCCTGTGAAGGAAGCTACCATGAAAATTATCTTTCTTTAAGCTT 845

Pepo-CD94-1   TCTTATTCCTTCAAGAAGTAAGATTTTGCGGT---TTTTTTTC-A-AAATAAATGGGCCTGATAAATGAG 903
Pepo-CD94-2   TCTTATTCCTTCAAGAAGTAAGATTTTGCGGT---TTTTTTTC-A-AAATAAATGGGCCTGATAAATGAG 903
Pepo-CD94-3   TTTTATTCCTTCAAGAAGTAAGATTTTGCGGTATTTTTTTTTCCATAAATAAATGGGCCTGATAAATGAG 915

Pepo-CD94-1   CAATTTGAGGCATCTGACTATACTTTCTAAATTAAAAGTTAAAAGTAATTTGTATGATGGACAAGCTGAA 973
Pepo-CD94-2   CAATTTGAGGCATCTGACTATACTTTCTAAATTAAAAGTTAAAAGTAATTTGTATGATGGACAAGCTGAA 973
Pepo-CD94-3   CAATTTGAGGCCTCTGACTATACTTTCTAAATTGGCAGTTAAAAGTAATTTGTATGATGGACAAGCTGAA 985

Pepo-CD94-1   TCTTATTCTGAATGCTGATGTCCTTAGAAAGATGTTGTACCCAGAAAATATTCAAATCAGCTAATATGTT 1043
Pepo-CD94-2   TCTTATTCTGAATGCTGATGTCCTTAGAAAGATGTTGTACCCAGAAAATATTCAAATCAGCTAATATGTT 1043
Pepo-CD94-3   TCTTATTCTGAACGCTCATGTCCTTAAAAAGACGTTGTACCCAGAAAATATTCAAATCAGCTAATATGTT 1055

                                 exon 5
Pepo-CD94-1   TGTCTCTGACTCTAAGCAGGCTTTTATGACCTCCAATCAAGAC 1086
Pepo-CD94-2   TGTCTCTGACTCTAAGCAGGCTTTTATGACCTCCAATCAAGAC 1086
Pepo-CD94-3   TGTCTCTGACTCTAAGCAGGCTTTTATGCCCTACAATCAACAG 1098
```

B

```
             exon 4
Tasy-CD94-1  CAGAAAAGTGGATTGGGTACCGATGCAACTGTTACTTCATTTCTAATGAGGAGAAAACTTGGGAAAAAAG 70
Tasy-CD94-2  CAGAAAAGTGGATTGGGTACCGATGCAACTGTTACTTCATTTCTAATGAGGAGAAAACTTGGGAAAAAAG 70
Tasy-CD94-3  CAGAGAAGTGGATTGGGTACCGATGCAACTGTTACTTCATTTCTAATGAGGAGAAAACTTGGGAAGGAAG 70
Tasy-CD94-4  CAGAGAAGTGGATTGGGTACCGATGCAACTGTTACTTCATTTCTAATGAGGAGAAAACTTGGGAAGGAAG 70
Tasy-CD94-5  CAGAGAAGTGGATTGGGTACCGATGCAACTGTTACTTCATTTCTAATGAGGAGAAAACTTGGGAAGGAAG 70
Tasy-CD94-6  CAGAGAAGTGGATTGGGTACCGATGCAACTGTTACTTCATTTCTAATGAGGAGAAAACTTGGGAAGGAAG 70
Tasy-CD94-7  CAGAGAAGTGGATTGGGTACCGATGCAACTGTTACTTCATTTCTAATGAGCAGAAAACTTGAGAAGAAAG 70
Tasy-CD94-8  CAGAGAAGTGGATTGGGTACCGATGCAACTGTTACTTCGTTTCTAATGAGAAGAAAACTTGGGAAGAAAG 70
Tasy-CD94-9  CAGAGAAGTGGATTGGGTACCGATGCAACTGTTACTTCGTTTCTAATGAGGAGAAAACTTGGGAAGAAAG 70

                                                                intron 4
Tasy-CD94-1  TAGGCAGTTCTGTGATTCTCTGAACTCCAGTCTACTTCAACTTCAAACCAGAGATGAACCGGCATGTACT 140
Tasy-CD94-2  TAGGCAGTTCTGTGATTCTCTGAACTCCAGTCTACTTCAACTTCAAACCAGAGATGAACCGGCATGTACT 140
Tasy-CD94-3  TAGGCAATTCTGTGCTTCTCTGAACTCCAGTCTACTTCAACTTCAAACCAGAGATGAACTGGCATGTACC 140
Tasy-CD94-4  TAGGCAATTCTGTGCTTCTCTGAACTCCAGTCTACTTCAACTTCAAACCAGAGATGAACTGGCATGTACC 140
Tasy-CD94-5  TAGGCAATTCTGTGCTTCTCTGAACTCCAGTCTACTTCAACTTCAAACCAGAGATGAACTGGCATGTACC 140
Tasy-CD94-6  TAGGCAATTCTGTGCTTCTCTGAACTCCAGTCTACTTCAACTTCAAACCAGAGATGAACTGGCATGTATC 140
Tasy-CD94-7  TAGGCAGTTCTGTGCTTCTCTCAACTCCAGTCTACTTCAACTTCAAATCAGAGATGAACCGGCATGCACT 140
Tasy-CD94-8  TAGGCAGTCCTGTGCTTCTCTGAACTCCAGTCTACTTCAACTTCAAACCAGAGATGAACTGGCATGTACT 140
Tasy-CD94-9  TAGGCAGTCCCGTGCTTCTCTGAACTCCAGTCTACTTCAACTTCAAACCAGAGATGAACTGGCATGTACT 140

Tasy-CD94-1  AAGTCTGGTTTCATACATTTT-TTTGACCTAGAAAAATATACTATCTAATTAAGCTAAATAATGTAGTTC 209
Tasy-CD94-2  AAGTCTGGTTTCATACATTTT-TTTGACCTAGAAAAATATACTATCTAATTAAGCTAAATAATGTAGTTC 209
Tasy-CD94-3  ACGTCTGGTGTCATACATTTTCTTTAACCTAGAAAAATATACTATCTAACTAAGCTAAATAATGTAGTTC 210
Tasy-CD94-4  AAGTCTGGTGTCATACATTTTCTTTAACCTAGAAAAATATACTATCTAACTAAGCTAAATAATGTAGTTC 210
Tasy-CD94-5  AAGTCTGGTGTCATACATTTTCTTTAACCTAGAAAAATATACTATCTAACTAAGCTAAATAATGTAGTTC 210
Tasy-CD94-6  AAGTCTGGTGTCATACATTTTCTTTAACCTAGAAAAATATACTATCTAACTAAGCTAAATAATGTAGTTC 210
Tasy-CD94-7  AAGTCTAAGTT-------TTTCTTTAACCTAGAAAAATATACTATCTAACTGAGCTAAATAATGTGGTTC 203
Tasy-CD94-8  AAGTCTGGTTTCCTACATTTTCTTTA-----GAAAAATATACTATCTAACTAAGCTAAATAATGTAGTTC 205
Tasy-CD94-9  AAGTCTGGTTTCCAACATTTTCTTTA-----GAAAAATATACTATATAACTAAGCTAACTAATGTAGTTC 205

Tasy-CD94-1  GAGTGTATTTTATTTTTT-----------GTGAGCTTGAGTCTCATTCTGTTGCCCAGGCTGGAGTCCAG 268
Tasy-CD94-2  GAGTGTATTTTATTTTTT-----------GTGAGCTTGAGTCTCATTCTGTTGCCCAGGCTGGAGTCCAG 268
Tasy-CD94-3  AAGTGTATTT-ATTTATTTATTTTTCTTTTTGAGCTTGAGTCTCACTCTGTTGCCCAGGCTGGAGTGCAG 279
Tasy-CD94-4  AAGTGTATTT-ATTTATTTATTTTTCTTTTTGAGCTTGAGTCTCACTCTGTTGCCCAGGCTGGAGTGCAG 279
Tasy-CD94-5  AAGTGTATTT-ATTTATTTATTTTTCTTTTTGAGCTTGAGTCTCACTCTGTTGCCCAGGCTGGAGTGCAG 279
Tasy-CD94-6  AAGTGTATTT-ATTTATTTATTTTTCTTTTTGAGCTTGAGTCTCACTCTGTTGCCCAGGCTGGAGTGCAG 279
Tasy-CD94-7  AAGTGTTTTTTTTCGTTTGTTTGTTTT----GAGCTTGAGTCGCACT-T-TTGCCCAGGCTGGAGTGCAG 267
Tasy-CD94-8  AAGTGTTTTTCTTTTTTTTTTTTT-------GAGCTTGAGTCTCACTCTGTTGCCCAGGCTGGATTGCAG 268
Tasy-CD94-9  AAGTGTATNNNNNNNNNNNNNNNNNNNNNNNGAGCTTGAGTCTCACTCTGTCGGCCAGGCTGGATTGCAG 275

Tasy-CD94-1  TGGCACACTCATGGCCACCCCAGTGTCGACCTGCTGGGCACTGACGATCCTTCTGCCTCAGCCTTTCAAG 338
Tasy-CD94-2  TGGCACACTCATGGCCACCCCAGTGTCGACCTGCTGGGCACTGACGATCCTTCTGCCTCAGCCTTTCAAG 338
Tasy-CD94-3  GGGCACACTCATGGACACCCCAGTGTCTACCTGCTGGGCACTGACGATCCTTCTGCCTTAGTCTTTCAAG 349
Tasy-CD94-4  GGGCACACTCATGGACACCCCAGTGTCTACCTGCTGGGCACTGACGATCCTTCTGCCTTAGTCTTTCAAG 349
Tasy-CD94-5  TGGCACACTCATGGACACCCCAGTGTCGACCTGCTGGGCACTGACGATCCTTCTGCCTTAGTCTTTCAAG 349
Tasy-CD94-6  TGGCACACTCATGGACACCCCAGTGTCGACCTGCTGGGCACTGACGATCCTTCTGCCTTAGTCTTTCAAG 349
Tasy-CD94-7  TGGCACACTCATGGCCACCCCAGTGTCGACCTGCTGGGCACTGACGATCATTCTGCCTCAGCCTTTCAAG 337
Tasy-CD94-8  TGGAACACTCATGGCCACCCCAGTGTCGACCTGCTGGGCACTGATGATCCTTCTGCCTCAGCCTTTCAAG 338
Tasy-CD94-9  TGGCACACTCATGGCCACCCCAGTGTCGACCTGCTGGGCACTGATGATCCTTCTGCCTCAGCCTTTCAAG 345

Tasy-CD94-1  TAGCTGCAATTTTGCGTGCATGCTACCACACCCGGCTAATTTTTGCTTTATCATAGAAACGAGGTCTCAC 408
Tasy-CD94-2  TAGCTGCAATTTTGCGTGCATGCTACCACACCCGGCTAATTTTTGCTTTATCATAGAAACGAGGTCTCAC 408
Tasy-CD94-3  TAGCTGGAATTTTGG                                                        364
Tasy-CD94-4  TAGCTGGAATTTTGG                                                        364
Tasy-CD94-5  TAGCTGGAATTTTGG                                                        364
Tasy-CD94-6  TAGCTGGAATTTTGG                                                        364
Tasy-CD94-7  CAGCTGGAATTTTGG                                                        352
Tasy-CD94-8  TACCTGGAATTTTG                                                         352
Tasy-CD94-9  TACCTGGAATTTTG                                                         359

Tasy-CD94-1  CAAATTGCCTGGGCTGGTCTTGAACCTGAAGGCTCCAGCAATTCTGTCACCTCGGCATCCCAAAGTGCTC 478
Tasy-CD94-2  CAAATTGCCTGGGCTGGTCTTGAACCTGAAGGCTCCAGCAATTCTGTCACCTCGGCATCCCAAAGTGCTC 478

Tasy-CD94-1  AGATTACAGGCTGAGCCACTGAGTCCAGCTTTATTCCAGTGTTTAATCAGATGGTAAATGAATGAACCAA 548
Tasy-CD94-2  AGATTACAGGCTGAGCCACTGAGTCCAGCTTTATTCCAGTGTTTAATCAGATGGTAAATGAATGAACCAA 548

Tasy-CD94-1  TGACCTATCAATTCCATGTCCCGTGAAATAATCAACATAAAACTTGCATAAAAGATCTTTAAGATTTATG 618
Tasy-CD94-2  TGACCTATCAATTCCATGTCCCGTGAAATAATCACCATACAACTTGCATAAAAGATCTTTAAGATTTATG 618
```

```
Tasy-CD94-1      TCATAGGAAAAAGATCAAGAGAATTACCAAATGAAGGCAAAAGAAAGTTTTCAGGGAGAATTCAAAGAAC 688
Tasy-CD94-2      TCATAGGAAAAAGATCAAGATAATTACCAAATGAAGGCAAAAGAAAGTTTTCAGGGAGAATTCAAAGAAC 688

Tasy-CD94-1      GAAACAATAGGTCATCATATTTACCCATTTTATTAAGGAAACTCAACTAAATTTCTTTTTGATTTTTAGC 758
Tasy-CD94-2      GAAACAATAGGTCATCATATTTACCCATTTTATTAAGGAAACTCAACTAAATTTCTTTTTGATTTTTAGC 758

Tasy-CD94-1      TATTTTAATGGAATTTATAATTTATTTTGTAATTTTACAGGTTTTGTCATTTTTCTGTTATATAATATGG 828
Tasy-CD94-2      TATTTTAATGGAATTTATAATTTATTTTGTAATTTTACAGGTTTTGTCATTTTTCTGTTATATAATATGG 828

Tasy-CD94-1      AACTAGTATGGAGATTATGATCATATAAATCACTCAATTCTTAAGAAAAAGTGAAAAACTTCTTCAGCTA 898
Tasy-CD94-2      AACTAGTATGGAGATTATGATCATATAAATCACTCAATTCTTAAGAAAAAGTGAAAAACTTCTTCAGCTA 898

Tasy-CD94-1      CATGCTAGCACCTGTGCTGACTATAGATTAAATAGATTTATCATTAAAATACTATTCATTTGGAATCAAA 968
Tasy-CD94-2      CATGCTAGCACCTGTGCTGACTATAGATTAAATAGATTTATCATTAAAATACTATTCATTTGGAATCAAA 968

Tasy-CD94-1      TTTGATGGTTGAATCTTGTGGAGGAAGTGTTTCATGTAAAGTCTACCTCCCTTTATAAGGCTTTTCTTTT 1038
Tasy-CD94-2      TTTGATGGTTGAATCTTGTGGAGGAAGTGTTTCATGTAAAGTCTACCTCCCTTTATAAGGCTTTTCTTTT 1038

Tasy-CD94-1      CTTCAAGAAGTGAGATTTTGTGTTATTTTTATCTTGCTAAATGAATGTGCCTGATCGATAAAAAATGTGA 1108
Tasy-CD94-2      CTTCAAGAAGTGAGATTTTGTGTTATTTTTATCTTGCTAAATGAATGTGCCTGATCGATAAAAAATGTGA 1108

Tasy-CD94-1      GGCCTGGACTATACCTTCTATATTAGAAATTAAAAATAAGTTCAGTTTGTGTAACGGAAAGGCTGAATTT 1178
Tasy-CD94-2      GGCCTGGACTATACCTTCTATATTAGAAATTAAAAATAAGTTCAGTTTGTGTAACGGAAAGGCTGAATTT 1178

Tasy-CD94-1      TATTCTAAATGCAAACTTTCTTTAGAACATGTTATTTCCAAGGTACTTAAAATTATATTACTTTGTTTTT 1248
Tasy-CD94-2      TATTCTAAATGCAAACTTTCTTTAGAACATGTTATTTCCAAGGTACTTAAAATTATATTACTTTGTTTTT 1248

                                 exon 5
Tasy-CD94-1      CTCTGTCTCTTTGCAGGATTTCATGCACTCCAGTCAGTAC 1288
Tasy-CD94-2      CTCTGTCTCTTTGCAGGATTTCATGCACTCCAGTCAGTAC 1288
```
