# Supplementary figures and images for: A Novel System of Polymorphic and Diverse NK Cell Receptors in Primates
Source: PLoS Genet. 2009 Oct 16;5(10):e1000688. doi: 10.1371/journal.pgen.1000688 (PMC2757895; doi:10.1371/journal.pgen.1000688)

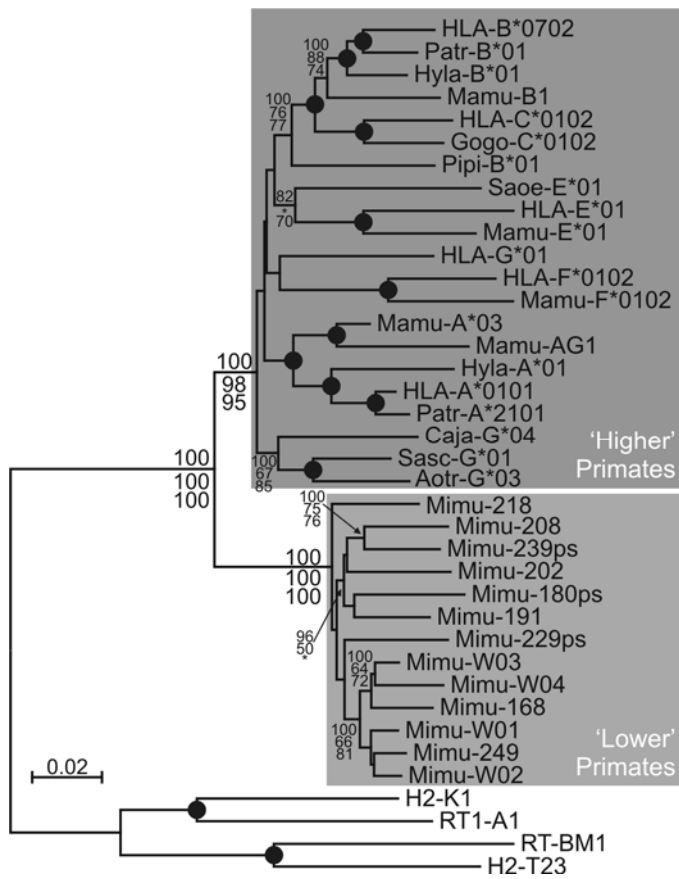

Supplement: Figure S5 — Phylogenetic analyses of MHC class I genes with complete coding sequences excluding the peptide binding residues. Analysis and display are as described for Figure 3. Numbers as gene names for MHC class I are the same as in Figure 1C. Non-primate sequences included are mouse (H2) and rat (RT1). Peptide binding residues were defined according to Bjorkman et al. [44]. Patr, Pan troglodytes; Gogo, Gorilla gorilla; Hyla, Hylobates lar; Saoe, Saguinus oedipus; Sasc, Saimiri sciureus; Mamu, Macaca mulatta; Mimu, Microcebus murinus; Aotr, Aotus trivirgatus; Caja, Callithrix jacchus; Pipi, Pithecia pithecia. (0.07 MB PDF) [file pgen.1000688.s005.pdf]

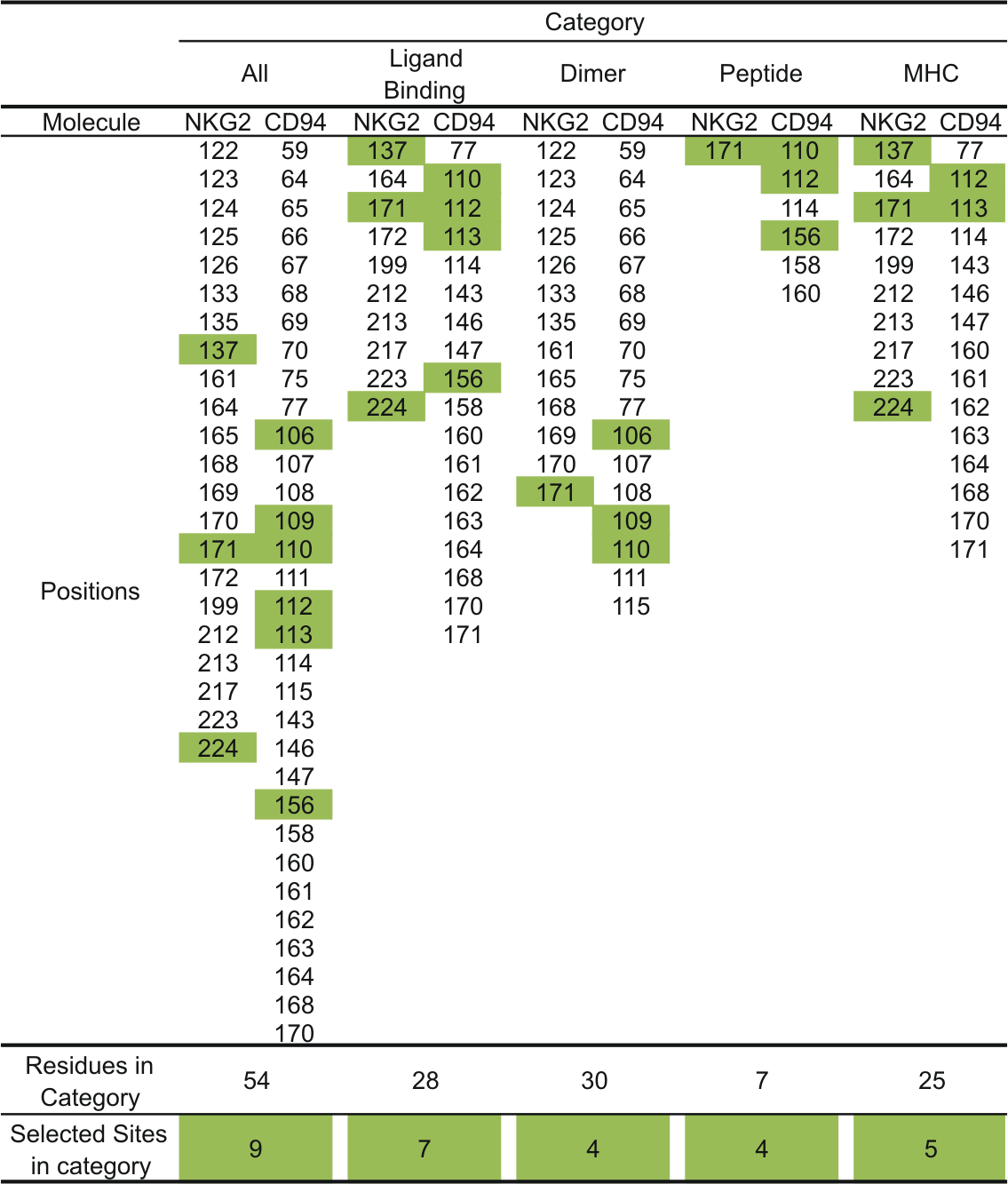

Supplement: Figure S6 — Contacts of CD94/NKG2 to bound peptide (Peptide) and to the MHC class I heavy chain (MHC) as well as residues mediating heterodimer formation (Dimer) based on the human CD94/NKG2A crystal structure [22],[23]. Additionally, Peptide and MHC contacts (Ligand binding) and all known contact residues (All) are summarised. (4.54 MB TIF) [file pgen.1000688.s006.tif]
