# Supplementary material for: A Novel System of Polymorphic and Diverse NK Cell Receptors in Primates
Source: PLoS Genet. 2009 Oct 16;5(10):e1000688. doi: 10.1371/journal.pgen.1000688 (PMC2757895; doi:10.1371/journal.pgen.1000688)
Supplement: Table S1 — Nucleotide and amino acid substitutions between different alleles of mouse lemur CD94, NKG2, and Ly49L sequences. SNPs were determined by sequencing the exons coding for the lectin-like domain (exon 4–6) of 12 individuals for NKG2 and Ly49L. For CD94 a further 34 animals were analysed. (0.04 MB PDF) [file pgen.1000688.s007.pdf]

Gene	Exon	Nucleotide position	Codon[a]	Codon number	Amino acids[b]
CD94-1	4	187	**S**AG	63	E/Q
		272	T**Y**T	91	S/F
	5	332	A**R**T	111	N/S
		376	**R**GA	126	G/R
		394	**R**AT	132	N/D
		403	**R**CT	135	A/T
	6	474	AC**R**	158	T
		527	C**W**A	176	L/Q
CD94-2	4	184	**R**AG	62	K/E
		190	**R**TT	64	V/I
		200	C**R**A	67	R/Q
		213	TA**Y**	71	Y
		222	TC**Y**	74	S
		262	**R**TT	88	I/V
		267	TC**D**	89	S
		299	A**R**A	100	R/K
	5	331	**Y**AC	111	Y/H
		362	A**S**A	121	T/R
		373	**R**AC	125	D/N
		400	**Y**TT	134	F/L
	6	455	G**Y**G	152	V/A
		463	**R**CA	155	A/T
		467	A**Y**G	156	M/T
		597	AC**S**	199	T
CD94-3	4	325	**W**AC	109	N/Y
		331	**R**AT	111	D/N
	5	354	TT**Y**	118	F
		396	GA**R**	132	E
	6	454	**Y**TG	152	L
		471	AC**R**	157	T
		474	AA**K**	158	K/N
		485	G**Y**G	162	V/A
		506	G**Y**T	169	A/V
		552	CA**R**	184	Q
		558	CT**Y**	186	L
		561	AT**Y**	187	I

NKG2-1	4	361	**R**AG	121	K/E
		431	A**Y**G	144	T/M
	6	593	A**Y**A	198	T/I
		599	A**Y**A	200	T/I
		673	**Y**TG	225	L
		685	**M**AG	229	K/Q
		695	C**K**T	232	R/L

NKG2-2	4	(345)	(TA**Y**)	(115)	(Y)
		366	GA**R**	122	E
		375	CT**Y**	125	L
		437	A**M**R	146	K/T
		438	AM**R**	146	K/T
	5	520	**R**CR	174	T/A
		522	RC**R**	174	T/A
		558	CC**V**	186	P
		561	TG**R**	187	W/Stop
		562	**R**TA	188	I/V
		566	T**Y**A	189	S/L
		576	GG**S**	192	G
	6	616	**R**RA	206	K/R/E/G
		617	R**R**A	206	K/R/E/G
		651	CT**K**	217	L
		661	**K**GA	221	G/Stop
		683	T**W**T	228	Y/F
		696	CA**Y**	232	H

NKG2-3	4	376	**R**CA	126	T/A
		424	**R**AT	142	N/D
		437	A**M**R	146	K/T
		438	AM**R**	146	K/T
		453	AA**V**	151	K/N
		477	GA**Y**	159	D
		484	**R**AA	162	K/E
		487	**R**AA	163	K/E
	5	516	AA**K**	172	K/N
		522	GC**R**	174	A
		558	CC**V**	186	P
		562	**R**TA	188	I/V
		566	T**Y**A	189	S/L
		576	GG**Y**	192	G

		591	CT**K**	197	L
	6	604	**W**CA	202	T/S
		607	**R**TA	203	V/I
		610	**Y**MT	204	H/P/S/Y
		611	Y**M**T	204	H/P/S/Y
		651	CT**K**	217	L
		657	TC**R**	219	S
		673	**V**CA	225	T/A/P
		683	T**W**T	228	Y/F
		697	**R**AG	233	K/E
		700	**Y**YT	234	L/P/S/F
		701	Y**Y**T	234	L/P/S/F
		704	T**R**G	235	W/Stop

NKG2-5	4	(335)	(G**R**C)	(112)	(D/G)
		(338)	(C**R**T)	(113)	(H/R)
		396	TT**S**	132	L/F
		411	GA**S**	137	E/D
		418	**M**TA	140	I/L
		445	**S**TK	149	V/L
		447	ST**K**	149	V/L
		460	**R**AT	154	N/D
		465	GA**W**	155	E/D
		469	**R**AA	157	K/E
	5	490	**Y**TT	164	L/F
		520	**Y**GT	174	R/C
		532	**R**RT	178	N/S/D/G
		533	R**R**T	178	N/S/D/G
		571	**M**TT	191	I/L
	6	587	A**Y**A	196	T/I
		589	**Y**YA	197	L/P/S
		590	Y**Y**A	197	L/P/S

NKG2-8	4	(354)	(GG**Y**)	(118)	(G)
		386	A**S**C	129	S/T
		390	AA**Y**	130	N
		398	T**M**C	133	Y/S
		402	AT**Y**	134	I
		403	**R**GT	135	S/G
		413	T**Y**C	138	S/F
		424	**R**AT	142	N/D

		438	AC**R**	146	T
		459	TC**Y**	153	S
		482	G**M**A	161	E/A
		484	**R**AA	162	K/E
		487	**R**AR	163	K/E
		489	RA**R**	163	K/E
		492	AT**D**	164	I/M
	5	558	CC**R**	186	P
		580	**M**CT	194	T/P
		593	C**M**A	198	Q/P
	6	598	**K**TA	200	V/L
		605	A**Y**A	202	T/I
		620	C**R**T	207	H/R
		629	G**Y**T	210	A/V
		634	**Y**TA	212	L
		646	**R**GA	216	R/G
		660	GA**K**	220	E/D
		662	G**R**A	221	E/G
		666	TG**Y**	222	C
		668	G**R**A	223	E/G
		674	T**Y**G	225	S/L
		684	TA**Y**	228	Y
		687	CA**Y**	229	H

Ly49L	4	(436)	(**Y**RT)	(146)	(H/R/Y/C)
		(437)	(Y**R**T)	(146)	(H/R/Y/C)
		487	**R**TG	163	M/V
		513	TG**Y**	171	C
		514	**R**AA	172	K/E
		526	**M**RA	176	R/Q
		527	M**R**A	176	R/Q
		533	T**W**C	178	Y/F
		562	**R**AA	188	K/E
		570	GA**R**	190	E
	5	574	**R**CC	192	T/A
		597	TA**Y**	199	Y
		642	AG**Y**	214	S
		675	CC**Y**	225	P
	6	702	TC**R**	234	S
		725	G**S**A	242	G/A
		811	**K**TC	271	V/F

		821	T**Y**A	274	S/L
		842	A**K**R	281	R/I/M
		843	AK**R**	281	R/I/M

NKG2D	4	268	**H**TA	90	L
		278	A**R**A	93	R/K
		377	A**R**A	126	A/G
	5	405	GT**S**	135	V
		429	CT**M**	143	L
		458	C**R**R	153	Q/R
		459	CR**R**	153	Q/R
		465	GA**M**	155	E/D
		471	GG**Y**	157	G
		474	AC**Y**	158	T
		480	CT**S**	160	L
	6	542	A**R**C	181	N/S
		572	A**Y**T	191	T/I
		600	AG**R**	200	R

[a] codons in parentheses indicate amino acids that belong to the stalk region and not the C-type lectin-like domain.
[b] as no haplotypes were deduced, the amino acids of the underlying SNPs could not be determined. In these cases all possible combinations are shown.
