# Supplementary material for: A Novel System of Polymorphic and Diverse NK Cell Receptors in Primates
Source: PLoS Genet. 2009 Oct 16;5(10):e1000688. doi: 10.1371/journal.pgen.1000688 (PMC2757895; doi:10.1371/journal.pgen.1000688)
Supplement: Table S2 — Primer sequences and performed PCRs. (0.08 MB PDF) [file pgen.1000688.s008.pdf]

primer	sequence
GeneRacer^{TM} 3′Primer GeneRacer Kit(Invitrogen)	GCTGTCAACGATACGCTACGTAACG
GeneRacer^{TM} 5’Primer GeneRacer Kit(Invitrogen)	CGACTGGAGCACGAGGACACTGA
Oligo(dT)-adapter primer	GACTCGAGTCGACATCGATTTTTTTTTTTTTTTTT
2697	GACTCGAGTCGACATCGA
3020	TCTCTGAATCACACAGCTGCA
3021	GACTCCAAAATCAGGTGCTTTA
3022	TCTGAGTCACACAGCTGCG
3023	TCAACTCCAAACTCAGGTGCT
3024	GTCTGACATCACACAGTTGCA
3025	GCTCCAAACTCAGGTGCTTTT
3026	TCCCTGACATCACACAGCTGT
3027	ACTCCCAACTCAGGTGCTTTC
3028	CCTGACATTACACAGCAGCG
3029	CCAACTCCAAAGTCAGACACT
3032	CCGTCTCTTTGTTCCAATAAAC
3033	GACCTTGGAACATCGGTTCA
3034	CACCTCTCTGACCCAAGG
3035	GACCTTGGAACATCGGTTCT
3037	TGGCCTTGGAACATCAGTTCT
3039	TGGCTTTTGTTGAGATGGTTG
3040	CAACAATGGACTAAGTCCTGC
3047	AAGATGAGTGATCAGGGAGTG
3215	AGTGGGAGGATGGCTCTAAT
3217	AGACAGTTTGACACATTTTACAG
3280	GTAATCTACTCTGAAATGAATCTG
3324	GCAGTGTCTCAGACCACTC
3325	GCAGTGTCTCAGACCACTA
3326	CATTTTACAGAACTAAGAAATAGATAATT
3327	AAGCTTGTGCTTACAATGGTATC
3328	CAGCAGCGGAGATGGATAAC
3329	AGGCTCGTGCTTACAAATATATG
3330	CAGTTGCAGAGATGGATAATCAA
3331	AAGCTTGTGCTTACAAATATATGT
3332	CAGCTGCGGAGATGAAGAA
3333	AAACTCATGCTTACAAATATATGT
3334	CAGCTGCAGAGATGAAGAA
3414	AAGATGTGTCATCCCCTCGAAGATCC
3417	GGAGTCTCTCGTAAAAGCCATCGTCA
3418	CCCAGCAATGAGCTTTTCTGGAGGTA
3461	GAGCTATAGCAATGGAACGGCAGAGA
3464	TCTGAGTGAGTCTAGTGCAGCACAGTGA
3465	GGAGCTTTTCCCGCCCTTAGATTTTG
3466	ATTGCTGGGCTCTCTGTCACTTCTGT
3467	GCTCCCTTTCAGGGAAAACATGGA
3471	CCCAATGTCATATGCAATGTTTTA
3472	CATGGAATTGATCGGTCATTAG
3478	TCATTTGTTCAAAAATAACTGTCCAT
3479	TTGTATGATGAGCAGGCTGAAT
3480	TTCATGACCACAAGCTCACC
3485	ACTTCTCTGGGCAAGAACAGCAGTCA
3488	GGTTTTCCATGAACAGCAAGA
3489	CATGGGTCATAATTTTCCTCATT

3490	GCAACATTTCACTAACTTCCAACA
3492	GATCCAGACACAAAGGCAATG
3495	TGCGTGTATGAGCGAGAATG
3496	GCATGGTTTTCCTTAAATAGCAA
3497	TTTGCCAATTACTGGTTACTCTT
3505	TTTCAACTTAATCTTGCTTAAAACAG
3506	TCCTGTACTTTCTGTTGTCTGCAT
3511	TGCCTGTATCAGCTTTCTGG
3512	GCATGGTTTTCTGTGAGAAGC
3513	TGAAAATTACAAACTTGTGCGAAA
3514	TGGTTGAGTGCCTTACTGTCA
3515	GCATCCACTGGTGTCTTGAA
3516	TCTGTTCTTTCTGGTGTGTGTATT
3531	TTTGCTATTCCAGAAAGGTGG
3588	CCACAAACGCAATGATGACC
3589	TGGTGGGCTTGAAACACATC
3590	TAAGGGGGACTGCTGTGGTT
3591	TACTTGTGAAATGTTTTGGCCAA
3625	GAGAGTCCAATCCAGTAAAA
3626	CTGACTGCTGTTCTTGCC
3628	CTCTGACTGCTGTTCTTGCC
3644	AGTTTGAGGGTATGGCATTCA
3645	CCAATAAAAGGAAACAAGTGTCG
3649	AACCACCAATAAAGGGAAACAA
3652	GGTCTTGAAACACAACCCCT
3656	GAAGTATGGTATACGCAGAATAG
3657	TTGTATCATAAGACAGTTTGACAC
3658	TCCAATGTCATTTGTGATATTTTATT
3659	TCCCTGTGATGTAAATCTTTGAA
3662	CTTCAGAAAAGTTCTAACTGCCT
3664	CTGTAGGAAGCCACAAATAAGTA
3668	TCGTGCACTTTATAATTTCAGATG
3702	GTGAATCTGCACCATTATGACTT
3703	AGCTTTGAGGATACATAGACATG
3734	CATGGGAATGCAGGTGTCTC
3735	GCACAACACGAACAACAGCT
3736	AGGAATGAAGGATGCTGCTG
3737	ACCAATGACTGCTACTTGAAAATG
3738	GGTTAACGCTTCCATATTCCAG
3739	TTCCTTTCTCTTCCAGCTCATC
3748	TGACCATGTCTTAGTGATCTGA
3749	TCCATGTTCATACTCCGGTG
3750	GGAATTATGCTAGTTAGGGAAGA
3751	TGCAACCAACACAATACCCTC
3752	GGCCAGACATGGTCCTAAAA
3753	TAAGAAATCTGATAGTCCGTCC
3758	GATGACAAAAGTTACAAGAGAAATTC
3759	GTTTCTTGTTCGTTTAATACGTGAG
3760	TTGTTATTTTGCCGCACCTTCTC
3761	ACATGAGATCTGTGTTTATTGAAAG
3762	GCACTTTATAATTTCAGATGTACATG
3854	GGAGCTAATATCCTCTCCAGGACTCA
3856	ATCACTCCAAACATCTCACTTTG
3857	CCTGTTCACATTAATAGCCACTG
3884	CAGAGATGAAGAACCAGAAAGT
3885	CACAAGCATCCAGATCTTCC
3887	TCAGTACACGTCTGCATTTTCT
3920	TCCAATGTCATTTGTGATGTTTTATT
4125	CCTGAGGATCTCTCTTCCTT
4126	CCCCGTCTCTTTGTTCCAAT

4128	TATGGCACCTGGCTAACTCC
4129	TGACATCACACAGCTGCAAAG
4130	GCTACAGGACGTTCTGGGGACAG
4141	TTGTTTCCCTTTATTGGTGGTT
4142	AGCAACGATATCCTGTTCTTTC
4143	CGAGGATGCAAAGATATGTACA
4144	TGCCCATTTTGTGATCTGACTTG
4361	AGAGTTGTTAGAAGCACAGGCCGTCA
4758	GGGTGTGGTAGCATGCAC
4851	ACGCTTGTGCTTACAAATATACAG
4932	ATTCCTTCACATCCAGCTCAG
4934	CCACTTTTCTGTCCCAAGAAG
4937	TATTCTGAGCACTGATGTTCTCA
4938	TCTGTGTTCTCAAGCCCTAAG
4939	CTACTGATGAGTAAGGGAATAAC
4940	CTATTCGGAGTAATCCTGGATC
4941	CTCAGTTTAATCTGTTTATCTCTG
4942	TTTAAAACTCCCTTGGCAGATC
4945	TACTTTATTTGGTGCCTATCTGAG
5026	TCCCAGGGTTATAACCTACTCA
5027	CAGAGAAGTGGGTTGGGTAC
5526	CTGTGGTGTCCACTGGTGT
5528	GATCCAGGCATCAGGTTGTT
5875	GCTCACAGATCCTCTTCTGAGATGAGTTTTTGTTCTTTGTAAT ACTGCCTCTGTGTG
6243	GTTCAGAGACCAGGGTCAGG
6244	CAACAGAAGTGGCACCATGT
6323	ACCATGGCAGTGTCTCAGACCACTA
6325	GCTTACTTGTCGTCATCGTCTTTGTAGTCAATAAGCTGTAGTA TACAGATG
6326	ACCATGGCAGTGTCTCAGACCACTC
6327	GCTTACTTGTCGTCATCGTCTTTGTAGTCATAGCAATAATGTG GGGTAAAAAT
6330	GCTTACTTGTCGTCATCGTCTTTGTAGTCAATGAGCTGTTGTT TACAGATGT

RT-PCR for amplification of mouse lemur NKC genes, DAP12 and KIR3DX1

PCR products were cloned into pGEM-T Easy vector and sequenced with vector primers.

PCR product	forward primer	reverse primer
Mimu-CD94-1 (cds)	3033	3032
Mimu-CD94-2 (3'-RACE)	3215	2697
Mimu-CD94-2 (cds)	3037	3217
Mimu-CD94-3 (cds)	3035	3034
Mimu-NKG2D (cds)	3885	3039
Mimu-NKG2-1 (cds)	3026	3027
Mimu-NKG2-2 (cds)	3884	3021
Mimu-NKG2-3 (cds)	3024	3025
Mimu-NKG2-5 (cds)	3022	3023
Mimu-NKG2-8 (cds)	3028	3029
Mimu-Ly49L (cds)	3047	3040
Mimu-DAP12 (cds)	5526	5528
Mimu-KIR3DX1 (cds)	6244	6243

cds, coding sequence; Mimu, *Microcebus murinus*

RT-PCR for generation of expression constructs

PCR products were cloned into expression vectors

PCR product	forward Primer	reverse Primer	expression vector
Mimu-CD94-1	3325	3032	pcDNA3.1/NT-GFP-TOPO
Mimu-CD94-2	3324	3326	pcDNA3.1/NT-GFP-TOPO
Mimu-CD94-3	3324	3034	pcDNA3.1/NT-GFP-TOPO
Mimu-NKG2-1	3026	4851	pcDNA3.1/V5-His-TOPO
Mimu-NKG2-2	3334	3333	pcDNA3.1/V5-His-TOPO
Mimu-NKG2-3	3330	3329	pcDNA3.1/V5-His-TOPO
Mimu-NKG2-5	3332	3331	pcDNA3.1/V5-His-TOPO
Mimu-NKG2-8	3328	3327	pcDNA3.1/V5-His-TOPO
Mimu-DAP12-Myc	5526	5875	pcDNA3.1/CT-GFP-TOPO
Mimu-CD94-1-Flag	6323	6325	pcDNA3.1/CT-GFP-TOPO
Mimu-CD94-2-Flag	6326	6327	pcDNA3.1/CT-GFP-TOPO
Mimu-CD94-3-Flag	6326	6330	pcDNA3.1/CT-GFP-TOPO

Mimu, *Microcebus murinus*

PCR to amplify CD94 sequences from the potto and the tarsier

PCR products were cloned into pGEM-T Easy vector and sequenced with vector primers.

PCR product	forward primer	reverse primer
Pepo-CD94-1	3626	3625
Pepo-CD94-2	3626	3625
Pepo-CD94-3	3626	3625
Pepo-CD94-4	3628	5026
Pepo-CD94-5	3628	5026
Pepo-CD94-6	5027	5026
Pepo-CD94-7	5027	5026
Tasy-CD94-1	3626	3625
Tasy-CD94-2	3626	3625
Tasy-CD94-3	3626	4758
Tasy-CD94-4	3626	4758
Tasy-CD94-5	3626	4758
Tasy-CD94-6	3626	4758
Tasy-CD94-7	3626	4758
Tasy-CD94-8	3626	4758
Tasy-CD94-9	3626	4758

Pepo, *Perodicticus potto*; Tasy, *Tarsius syrichta*

Amplification and sequencing of mouse lemur CD94, NKG2 and Ly49L (exon 4, 5 and 6)

For some individuals different primer pairs have been used for exon amplification (PCR1, PCR 2). In case of CD94-3 exon 4 for some individuals a nested PCR has been performed in which Mimu-CD94-3 exon 4 (PCR 1) served as template.

**PCR product**	**forward primer**	**reverse primer**	**sequencing primer**
Mimu-CD94-1 exon 4	3658	3659	3658, 3659, 3478
Mimu-CD94-1 exon 5 (PCR 1)	3479	3480	3479, 3480
Mimu-CD94-1 exon 5 (PCR 2)	4937	4938	4937, 4938
Mimu-CD94-1 exon 6 (PCR 1)	4125	4126	4125, 4126
Mimu-CD94-1 exon 6 (PCR 2)	4939	4940	4939, 4940
Mimu-CD94-2 exon 4	3471	3472	3471, 3472, 3664
Mimu-CD94-2 exon 5-6 (PCR 1)	3656	3657	3656, 3657
Mimu-CD94-2 exon 5 (PCR 2)	4941	4942	4941, 4942
Mimu-CD94-3 exon 4 (PCR 1)	3662	3758	3920, 3664
Mimu-CD94-3 exon 4 (nested PCR)	3857	4945	3857, 4945
Mimu-CD94-3 exon 5	3759	3760	3759, 3760
Mimu-CD94-3 exon 6	3761	3668	3761, 3668, 3856, 3762
Mimu-NKG2-1 exon 4	3531	3488	3531, 3488
Mimu-NKG2-1 exon 5	3489	3490	3489, 3490
Mimu-NKG2-1 exon 6	3652	3492	3652, 3492
Mimu-NKG2-2 exon 4	3495	3496	3495, 3496
Mimu-NKG2-2 exon 5	3497	3649	3497, 3649
Mimu-NKG2-2 exon 6	4141	4142	4141, 4142
Mimu-NKG2-3 exon 4	4143	4144	4143, 4144
Mimu-NKG2-3 exon 5	3505	3645	3505, 3645
Mimu-NKG2-3 exon 6	3644	3506	3644, 3506
Mimu-NKG2-5 exon 4	3511	3512	3511, 3512
Mimu-NKG2-5 exon 5	3513	3514	3513, 3514
Mimu-NKG2-5 exon 6	3515	3516	3515, 3516
Mimu-NKG2-8 exon 4	3702	3703	3702, 3703
Mimu-NKG2-8 exon 5	3590	3591	3590, 3591
Mimu-NKG2-8 exon 6	3588	3589	3588, 3589
Mimu-Ly49L exon 4	3734	3735	3734, 3735
Mimu-Ly49L exon 5	3736	3737	3736, 3737
Mimu-Ly49L Exon 6	3738	3739	3738, 3739
Mimu-NKG2D Exon 4	3748	3749	3748, 3749
Mimu-NKG2D Exon 5	3750	3751	3750, 3751
Mimu-NKG2D Exon 6	3752	3753	3752, 3753

Mimu, *Microcebus murinus*

RT-PCR and RACE for amplification of ruffed lemur NKC genes

PCR products were cloned into pGEM-T Easy vector and sequenced with vector primers.
Vava, *Varecia variegata*

Vava-CD94-1

	forward primer	reverse primer
RT-PCR	4932	4934
5'-RACE	GeneRacer™ 5'Primer	3485
3'-RACE	3854	GeneRacer™ 3'Primer

Vava-CD94-2

	forward primer	reverse primer
RT-PCR	3037	3217

Vava-CD94-3

	forward primer	reverse primer
RT-PCR	3035	3034
5'-RACE	GeneRacer™ 5'Primer	3485

Vava-NKG2D

	forward primer	reverse primer
RT-PCR	3887	3039
5'-RACE	GeneRacer™ 5'Primer	3461

Vava-NKG2-1

	forward primer	reverse primer
RT-PCR	3330	3329
5'-RACE	GeneRacer™ 5'Primer	3414
3'-RACE	3417	GeneRacer™ 3'Primer

Vava-NKG2-2

	forward primer	reverse primer
5'-RACE	GeneRacer™ 5'Primer	3464
3'-RACE	4130	GeneRacer™ 3'Primer

Vava-NKG2-3

	forward primer	reverse primer
RT-PCR	4129	4128
5'-RACE	GeneRacer™ 5'Primer	3418
3'-RACE	3467	GeneRacer™ 3'Primer

Vava-NKG2-4

	forward primer	reverse primer
RT-PCR	3020	3021
5'-RACE	GeneRacer™ 5'Primer	3464

Vava-NKG2-5

	forward primer	reverse primer
RT-PCR	3280	3027
5'-RACE	GeneRacer™ 5'Primer	4361

Vava-NKG2-6

	forward primer	reverse primer
RT-PCR	3020	3021

Vava-NKG2-7

	forward primer	reverse primer
RT-PCR	3020	3021
5'-RACE	GeneRacer™ 5'Primer	3465
3'-RACE	3466	GeneRacer™ 3'Primer

Vava-NKG2-8

	forward primer	reverse primer
RT-PCR	3280	3027
5'-RACE	GeneRacer™ 5'Primer	3418

Vava-Ly49L

	forward primer	reverse primer
RT-PCR	3047	3040
